# Synthesis with Perfect Atom Economy: Generation of Furan Derivatives by 1,3-Dipolar Cycloaddition of Acetylenedicarboxylates at Cyclooctynes

**DOI:** 10.3390/molecules190914022

**Published:** 2014-09-05

**Authors:** Klaus Banert, Sandra Bochmann, Andreas Ihle, Oliver Plefka, Florian Taubert, Tina Walther, Marcus Korb, Tobias Rüffer, Heinrich Lang

**Affiliations:** 1 Organic Chemistry, Technische Universität Chemnitz, Strasse der Nationen 62, 09111 Chemnitz, Germany; E-Mails: sandra.bochmann@chemie.tu-chemnitz.de (S.B.); andreas.ihle@chemie.tu-chemnitz.de (A.I.); oliver.plefka@web.de (O.P.); florian.taubert@s2008.tu-chemnitz.de (F.T.); tina.walther@s2008.tu-chemnitz.de (T.W.); 2 Inorganic Chemistry, Technische Universität Chemnitz, 09107 Chemnitz, Germany; E-Mails: marcus.korb@s2007.tu-chemnitz.de (M.K.); tobias.rueffer@chemie.tu-chemnitz.de (T.R.); heinrich.lang@chemie.tu-chemnitz.de (H.L.)

**Keywords:** cascade reactions, cyclopropenes, Diels–Alder reactions, dipolar intermediates, epoxides, orthoesters, oxygen heterocycles, reaction mechanisms, retro-Brook reactions, strained compounds

## Abstract

Cyclooctyne and cycloocten-5-yne undergo, at room temperature, a 1,3-dipolar cycloaddition with dialkyl acetylenedicarboxylates **1a**,**b** to generate furan-derived short-lived intermediates **2**, which can be trapped by two additional equivalents of **1a**,**b** or alternatively by methanol, phenol, water or aldehydes to yield polycyclic products **3b**–**d**, orthoesters **4a**–**c**, ketones **5** or epoxides **6a**,**b**, respectively. Treatment of bis(trimethylsilyl) acetylenedicarboxylate (**1c**) with cyclooctyne leads to the ketone **7** via retro-Brook rearrangement of the dipolar intermediate **2c**. In all cases, the products are formed with perfect atom economy.

## 1. Introduction

Esters of acetylene dicarboxylic acid and especially dimethyl acetylenedicarboxylate (DMAD, **1a**) are highly versatile tools for organic chemists [1,2,3,4,5,6,7]. These compounds are successfully utilized as dienophiles in Diels–Alder reactions, as dipolarophiles in 1,3-dipolar cycloadditions and also as components in [2 + 2] or other cycloaddition reactions. Furthermore, they can be used as powerful Michael acceptors, and in several cases, nucleophilic addition and formation of zwitterions are combined with other addition or (formal) cycloaddition steps to yield a variety of products by multicomponent reactions [1].

**Scheme 1:**
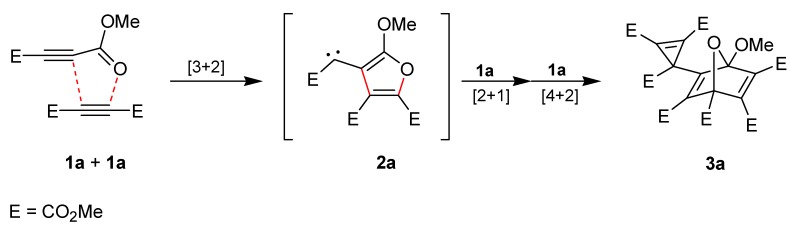
Tetramerization of dimethyl acetylenedicarboxylate (**1a**).

Tetramerization of **1a**, that has been performed by storing the neat substance at room temperature for several years or by heating it neatly or in solution, indicates an unusual reaction course of acetylenedicarboxylates (Scheme 1) [8,9,10]. Formation of the crystalline product **3a** was explained by a dimerization step, in which **1a** functions as dipolarophile and, also, as 1,3-dipole [8,10,11]. The resulting carbene intermediate **2a** is trapped by a third molecule of **1a**, and, finally, Diels–Alder reaction of the furan unit with a fourth molecule of **1a** leads to **3a**. The structure of this tetramer was confirmed by X-ray single crystal structure analysis [10,12]. The compound **3a** has been utilized in several transformations, such as thermal retro-Diels–Alder reactions [9,13] or [4 + 2]-cycloaddition in the presence of cyclopentadienes [14], as well as photolysis [8,9] or treatment with triphenylphosphine [10]. The tetramer of diethyl acetylenedicarboxylate has also been prepared by a procedure, which is analogous to the synthesis of **3a** [9]. However, formation of products with structures similar to that of **3a** is very limited when **1a** was heated with other alkynes or alkenes [13,15]. For example, the carbene intermediate **2a** can also be trapped in the presence of electron-poor dimethyl fumarate to generate the cyclopropane-derived compound dihydro-**3a**, but with an excess of tolane, the corresponding interception product was obtained only in trace amounts. This is remarkable because it is well known that carbenes, which were produced by photolysis of methyl aryl(diazo)acetate, react with acceptor-substituted and also electron-rich π-systems to form three-membered rings [16]. In a rhodium-catalyzed transformation, **1a** and terminal alkenes led to cyclopropane derivatives that might be explained by trapping of carbene **2a** with the help of the olefin [17,18]. However, such a mechanism was left out of consideration.

## 2. Results and Discussion

**Scheme 2:**
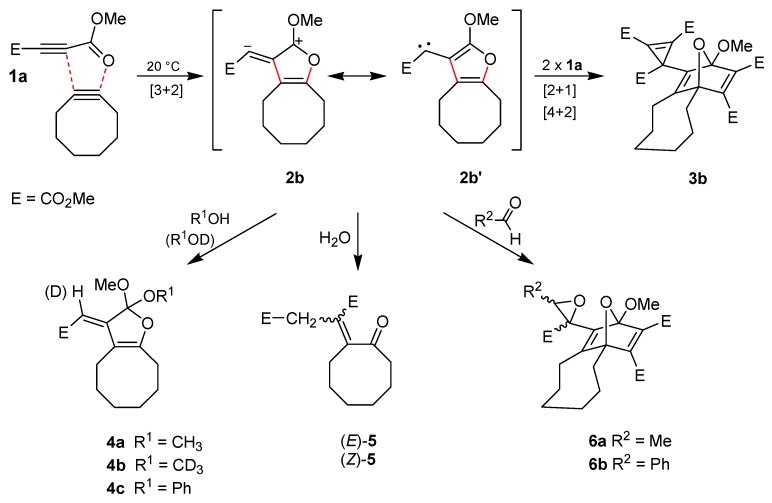
Reaction of **1a** with cyclooctyne in the absence or in the presence of other reagents.

**Figure 1:**
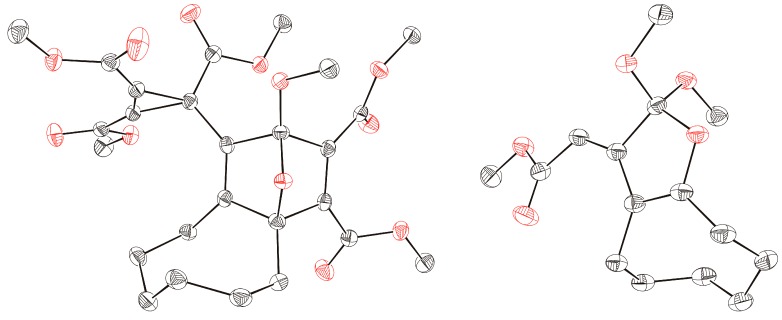
ORTEP representation (50% probability level) of the molecular structures of products **3b** (**left**) and **4a** (**right**); hydrogen atoms are omitted for clarity.

We accidentally found that the alkynes **1a** and cyclooctyne [19] undergo an exothermic reaction at room temperature [20,21,22,23]. Thus, the transformation was conveniently performed in dichloromethane (20 h/20 °C) and led to the crystalline product **3b** with 79% yield (Scheme 2). The structure of **3b** was confirmed not only by NMR spectroscopic data but also by single crystal X-ray diffraction analysis (Figure 1). Obviously, the 1,3-dipolar cycloaddition of **1a** at the ring-strained dipolarophile cyclooctyne to generate the intermediate **2b** is much more rapid than the dimerization of **1a** to produce **2a**. This allows the synthesis of the interception product **3b** without heating or long reaction times and also formation of several other trapping products of **2b**. 

After treatment of cyclooctyne with **1a** in methanol instead of dichloromethane, the orthoester **4a** was isolated in 60% yield. The surprising structure and especially the stereochemistry of this product were proved by spectroscopic data and X-ray diffraction analysis (Figure 1). When **1a** and cyclooctyne were analogously reacted in an excess of deuterated methanol (CD_3_OD), the product **4b**, which indicated the selective incorporation of exactly one equivalent of the deuterated reagent, was obtained with 62% yield. In the presence of phenol instead of methanol, the conversion of **1a** and cyclooctyne only led to a low yield (10%) of the corresponding orthoester **4c**. We did not get any orthoester trapping product with a structure similar to that of **4** after heating **1a** alone in pure methanol (up to 80 °C) because addition of the solvent at the C≡C bond to form dimethyl 2-methoxybut-2-enedioates dominated.

In the case of **4a**–**c**, we exclusively isolated the depicted (*E*)-stereoisomer, whereas a mixture of (*E*)**-5** and (*Z*)**-5** resulted after exposure of cyclooctyne to **1a** in aqueous tetrahydrofuran. The latter products were separated by chromatography to yield (*E*)**-5** (21%) and (*Z*)**-5** (20%), which were assigned with the help of NOE-NMR experiments. The genesis of **5** can be explained through interception of dipolar intermediate **2b** by water. The product of this step is similar to orthoesters **4** but includes the substructure of a cyclic hemiacetal, which is transformed into **5** by ring opening followed by tautomerism.

**Figure 2:**
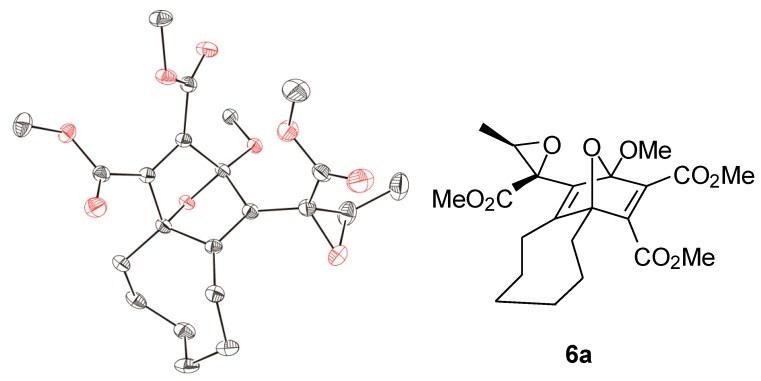
ORTEP representation (30% probability level) of the molecular structure of **6a**; hydrogen atoms are omitted for clarity; only one of the two enantiomers of the asymmetric unit is shown.

When aldehydes such as acetaldehyde or benzaldehyde were used as solvents for the reaction of cyclooctyne with **1a**, epoxides **6a** and **6b**, respectively, were formed (Scheme 2). In the case of **6a**, the ^1^H-NMR spectrum of the crude reaction mixture indicated the generation of two diastereomers in a ratio of about 3.5:1 although four diastereomers are possible. Only the main product, however, could be isolated by chromatography (22% yield), and the relative configurations of its stereocenters were determined by single crystal X-ray diffraction analysis (Figure 2). The epoxide **6b** was obtained in 21% yield as a mixture of two diastereomers, which could be separated by chromatography.

**Scheme 3:**
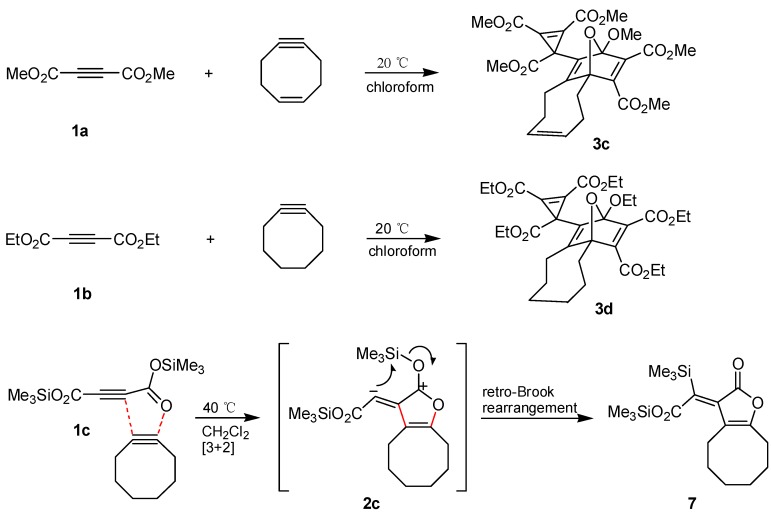
Furan derivatives from acetylenedicarboxylates and cyclooctynes.

The synthesis of **3b** from **1a** and cyclooctyne can be transferred to other acetylenedicarboxylates and cycloalkynes. Thus, **1a** underwent a similar cascade of cycloaddition reactions in the presence of cycloocten**-**5**-**yne [24,25,26] to afford the product **3c** in 37% yield (Scheme 3). On the other hand, diethyl acetylenedicarboxylate (**1b**) reacted only slightly slower than **1a** with cyclooctyne to give the polycycle **3d** with 61% yield. We attempted to perform the analogous transformation with di-*tert*-butyl acetylenedicarboxylate, however, not any characterizable product was obtained, even on heating the reaction mixture to 40 °C or on utilizing methanol as solvent. We assume that the sterically hindered diester is not able to enter into the rate-determining first step, which prevents not only the generation of the intermediate **2** but also the formation of products of type **3** or **4**. However, treatment of bis(trimethylsilyl) acetylenedicarboxylate (**1c**) with cyclooctyne in anhydrous dichloromethane at 40 °C led to the oily 1:1 adduct **7** (85% yield). The ^13^C, ^1^H long-range correlation 2D-NMR spectra and especially the ^29^Si**-**NMR data indicated that the structure of **7** includes an oxygen-bound trimethylsilyl group and also such a group directly connected with carbon (Scheme 3). The genesis of **7** is easily explained by a 1,3**-**dipolar cycloaddition of the substrates and subsequent retro-Brook rearrangement of the intermediate **2c**. This means that the corresponding vinylic carbon atom of **2c** should possess nucleophilic properties to allow an intramolecular attack at silicon. Thus, the question arises whether intermediates of type **2** have to be generally described by dipolar resonance structures such as **2b** and **2c** or by carbene structures like **2a** and **2b'**. The formation of the three-membered ring in the products **3a**–**d** and **6a**,**b**, respectively, can be interpreted via nucleophilic attack of the negatively charged carbon atom of **2** at the π-system of **1** or the aldehydes followed by ring closure of the resulting dipolar species or alternatively by a cheletropic reaction of the carbene version of **2** [27]. Furthermore, generation of orthoesters **4a**–**c** is possible through trapping of dipole **2b** and is less easily explained via carbene **2b'**. Finally, the retro-Brook rearrangement **2c**→**7** and also the fact that interception of **2** to prepare products with three-membered rings is successful only in case of electron-poor π-systems but not with simple alkenes or alkynes, are strong arguments to prefer the dipolar resonance structure of intermediates **2** [28,29,30,31,32].

## 3. Experimental

### 3.1. General Information

Melting points were determined with a Pentakon Dresden Boetius apparatus. FTIR spectra were recorded with a Nicolet iS5 spectrophotometer (Thermo Scientific) and solutions in KBr cuvettes. Alternatively, a FTS-165 spectrometer (BioRad) was used. ^1^H-NMR spectra were recorded with a Unity Inova 400 spectrometer operating at 400 MHz. By using the same spectrometer, ^13^C-NMR data were recorded at 100.6 MHz, ^2^H-NMR spectra were measured at 61.4 MHz and ^29^Si-NMR spectra at 79.5 MHz. NMR signals were referenced to TMS (δ = 0) or solvent signals and recalculated relative to TMS. The multiplicities of ^13^C-NMR signals were determined with the aid of DEPT135 experiments. HRMS (ESI) spectra were recorded with a Bruker micrOTOF-QII spectrometer. Elemental analyses were performed with a Vario Micro Cube from Elementar. HPLC was carried out with HPLC Pump 64 and Variable Wavelength Monitor (Knauer). TLC was performed with Macherey**-**Nagel Polygram SIL G/UV_254_ polyester sheets. Diffraction data for **3b**, **4a** and **6a** were collected with an Oxford Gemini S diffractometer, with graphite-monochromated Mo Kα radiation (λ = 0.71073 Å) (**3b**) or Cu Kα radiation (λ = 1.54184 Å) (**4a**, **6a**). The structures were solved by direct methods and refined by full-matrix least-squares procedures on *F*^2^ [33,34]. Graphics of the molecular structures have been created by using ORTEP [35]. All non-hydrogen atoms were refined anisotropically, and a riding model was employed in the treatment of the hydrogen atom positions.

### 3.2. Synthesis of Trimethyl 3-[2-Methoxy-3,4-bis(methoxycarbonyl)-5,6,7,8,9,10-hexahydro-2H-2,4a-epoxybenzo[8]annulen-1-yl]cycloprop-1-ene-1,2,3-tricarboxylate *(**3b**)*

To a solution of **1a** (1.00 g, 7.04 mmol, freshly distilled) in anhydrous CH_2_Cl_2_ (2.5 mL), cyclooctyne (189 mg, 1.75 mmol) was added with stirring at 0 °C. After 20 h at ambient temperature, the solvent and the excess of **1a** were removed at reduced pressure (finally 1 h at 40 °C and 0.1 mbar), and the residue was purified by flash chromatography (SiO_2_, CHCl_3_/ethyl acetate 15:1) to give **3b** (0.74 g, 79%) as a pale yellow solid, which was repeatedly crystallized from cyclohexane to yield colorless crystals with m.p. 90 °C that are appropriate for X-ray diffraction analysis. ^1^H**-**NMR (CDCl_3_): δ = 1.15–1.55 (m, 4H), 1.58–1.82 (m, 4H), 1.97–2.08 (m, 2H, 10'**-**H or 5'**-**H), 2.42‒2.62 (m, 2H, 10'-H or 5'-H), 3.52 (s, 3H, O-C-OCH_3_), 3.64 (s, 3H, OCH_3_), 3.75 (s, 3H, OCH_3_), 3.76 (s, 3H, OCH_3_), 3.84 (s, 3H, OCH_3_), 3.85 (s, 3H, OCH_3_). ^13^C-NMR (CDCl_3_): δ = 23.12 (t), 23.26 (t, 2C), 25.33 (t), 25.61 (t), 29.03 (t), 32.27 (s, C-3), 52.15 (q, 2C, OCH_3_), 52.75 (q, OCH_3_), 53.14 (q, OCH_3_), 53.17 (q, OCH_3_), 54.87 (q, O-C-O*C*H_3_), 89.42 (s, C-4a'), 114.19 (s), 115.65 (s, O-C-OCH_3_), 116.49 (s), 140.36 (s), 150.82 (s), 157.01 (s), 157.12 (s), 157.36 (s), 160.62 (s), 163.23 (s), 164.18 (s), 170.19 (s). IR (CDCl_3_): = 2954 (m), 1732 (s), 1268 (s) cm^−1^. Elemental Analysis calcd. for C_26_H_30_O_12_: C: 58.42%, H: 5.66%. Found: C: 58.45%, H: 5.63%.

*Crystal Data for* **3b**: C_26_H_30_O_12_, M = 534.50 g·mol^−1^, monoclinic, *C*2/*c*, λ = 0.71073 Å, *a* = 31.6876 (13) Å, *b* = 10.1200 (3) Å, *c* = 16.1580 (6) Å, β = 101.909 (4) °, *V* = 5070.0 (3) (4) Å^3^, *Z* = 8, ρ_calcd_ = 1.400 Mg∙m^−3^, μ = 0.112 mm^−1^, T = 100 (2) K, *θ* range 3.23°–25.00°, 9688 reflections collected, 4448 independent reflections (*R*_int_ = 0.0204), R1 = 0.0462, *w*R2 = 0.0865 (I > 2σ(I)).

### 3.3. Synthesis of Trimethyl 3-[2-Methoxy-3,4-bis(methoxycarbonyl)-5,6,9,10-tetrahydro-2H-2,4a-epoxybenzo[8]annulen-1-yl]cycloprop-1-ene-1,2,3-tricarboxylate *(**3c**)*

To cycloocten-5-yne (74 mg, 0.70 mmol) in CDCl_3_ (0.7 mL), **1a** (400 mg, 2.79 mmol) was added. The mixture was allowed to stand at room temperature for 20 h. After removal of the solvent at reduced pressure, the residue was purified by chromatography (silica gel, dichloromethane/ethyl acetate 15:1) to give **3c** (138 mg, 37%) as a slightly yellow oil. ^1^H-NMR (CDCl_3_): δ = 1.99–2.32 (m, 4H, CH_2_), 2.64–2.94 (m, 4H, CH_2_), 3.45 (s, 3H, O-C-OCH_3_), 3.61 (s, 3H, OCH_3_), 3.73 (s, 3H, OCH_3_), 3.77 (s, 3H, OCH_3_), 3.83 (s, 3H, OCH_3_), 3.84 (s, 3H, OCH_3_), 5.47–5.54 (m, 2H, CH=CH). ^13^C-NMR (CDCl_3_): δ = 24.03 (t), 24.07 (t), 27.55 (t), 27.64 (t), 31.53 (s), 52.03 (q, OCH_3_), 52.16 (q, OCH_3_), 52.63 (q, OCH_3_), 52.96 (q, OCH_3_), 53.07 (q, OCH_3_), 54.62 (q, O-C-O*C*H_3_), 91.06 (s, C-4a'), 113.51 (s), 115.23 (s, O-C-OCH_3_), 115.47 (s), 128.18 (d, CH=CH), 129.35 (d, CH=CH), 142.64 (s), 150.62 (s), 156.81 (s), 156.90 (s), 157.25 (s), 159.43 (s), 162.99 (s), 164.16 (s), 170.07 (s). IR (CDCl_3_): = 2954 (m), 1732 (s), 1270 (s) cm^−1^. HRMS: *m*/*z* calcd. for C_26_H_28_O_12_ ([M+H]^+^): 533.1659, found: 533.1592; calcd. for ([M+Na]^+^): 555.1478, found: 555.1470; calcd. for ([M+K]^+^): 571.1218, found: 571.1169.

### 3.4. Synthesis of Triethoxy 3-[2-Ethoxy-3,4-bis(ethoxycarbonyl)-5,6,7,8,9,10-hexahydro-2H-2,4a-epoxybenzo[8]annulen-1-yl]cycloprop-1-ene-1,2,3-tricarboxylate *(**3d**)*

To cyclooctyne (20 mg, 0.19 mmol) in CDCl_3_ (0.7 mL), **1b** (0.13 g, 0.74 mmol) was added. The mixture was allowed to stand at room temperature for 20 h. After removal of the solvent at reduced pressure, the residue was purified by chromatography (silica gel, dichloromethane/ethyl acetate 15:1) to give **3d** (70 mg, 61%) as a slightly yellow oil. ^1^H-NMR (CDCl_3_): δ = 1.13–1.20 (m, 6H, CH_3_), 1.25–1.35 (m, 12H, CH_3_), 1.39–1.83 (m, 8H, CH_2_), 2.01–2.12 (m, 2H, CH_2_), 2.48–2.67 (m, 2H, CH_2_), 3.70–3.78 (m, 1H, OCH_2_), 3.84–3.91 (m, 1H, OCH_2_), 3.99–4.08 (m, 1H, OCH_2_), 4.11–4.25 (m, 5H, OCH_2_), 4.27–4.36 (m, 4H, OCH_2_). ^13^C**-**NMR (CDCl_3_): δ = 13.99 (q, 2 C, CH_3_), 13.94 (q, CH_3_), 13.98 (q, CH_3_), 14.07 (q, CH_3_), 15.02 (q, CH_3_), 23.27 (t, CH_2_), 23.31 (t, CH_2_), 23.33 (t, CH_2_), 25.46 (t, CH_2_), 25.68 (t, CH_2_), 29.02 (t, CH_2_), 32.36 (s, C-3), 61.01 (t, OCH_2_), 61.08 (t, OCH_2_), 61.53 (t, OCH_2_), 62.22 (t, OCH_2_), 62.37 (t, OCH_2_), 63.59 (t, OCH_2_), 89.29 (s, C**-**4a'), 114.65 (s), 115.40 (s), 116.20 (s), 140.68 (s), 151.17 (s), 156.87 (s), 156.91 (s), 157.17 (s), 160.07 (s), 162.96 (s), 163.91 (s), 169.68 (s). IR (CDCl_3_): = 2984 (m), 1727 (s), 1260 (s) cm^−1^. HRMS: *m*/*z* calcd. for C_32_H_42_O_12_ ([M+H]^+^): 619.2755, found: 619.2697; calcd. for ([M+Na]^+^): 641.2574, found: 641.2608; calcd. for ([M+K]^+^): 657.2313, found: 657.2263.

### 3.5. Synthesis of Methyl (E)-2-(2,2-Dimethoxy-4,5,6,7,8,9-hexahydrocycloocta[b]furan-3(2H)-ylidene)acetate *(**4a**)*

To cyclooctyne (271 mg, 2.51 mmol) in anhydrous MeOH (2.5 mL), **1a** (1.40 g, 9.85 mmol) was added. The mixture was stirred at room temperature for 20 h. After removal of the solvent at reduced pressure, the residue was purified by chromatography (silica gel, cyclohexane/*tert*-butyl methyl ether 14:1) to give **4a** (426 mg, 60%) as a colorless solid with m.p. 60–61 °C (from cyclohexane/*tert***-**butyl methyl ether). ^1^H**-**NMR (CDCl_3_): δ = 1.39–1.46 (m, 2H, 6'**-**CH_2_), 1.49–1.57 (m, 2H, 7'**-**CH_2_), 1.61–1.69 (m, 2H, 5'**-**CH_2_), 1.68–1.75 (m, 2H, 8'**-**CH_2_), 2.46 (m, 2H, 9'**-**CH_2_), 2.73 (t, *J* = 6.3, 2H, 4'**-**CH_2_), 3.33 (s, 6H, 2'**-**(OCH_3_)_2_), 3.69 (s, 3H, CO_2_CH_3_), 5.50 (s, 1H, 2**-**CH). ^13^C**-**NMR (CDCl_3_): δ = 22.2 (t, C**-**4'), 25.6 (t, C**-**6'), 26.5 (t, C-7'), 27.1 (t, C-9'), 28.7 (t, C**-**8'), 29.3 (t, C-5'), 51.0 (q, 1-OCH_3_), 51.3 (q, 2'-(OCH_3_)_2_), 106.3 (d, C-2), 112.5 (s, C-3a'), 121.7 (s, C-2'), 152.1 (s, C-3'), 166.3 (s, C-1), 170.3 (s, C-9a'). IR (film): = 2928 (s), 2853 (m), 1718 (s), 1642 (m), 1595 (s), 1458 (m), 1441 (m), 1353 (m), 1324 (m), 1277 (s), 1226 (s), 1167 (s), 1126 (s), 1086 (m), 1050 (s), 1022 (m), 1009 (m), 969 (m), 931 (m), 911 (m), 886 (w), 843 (m), 778 (w) cm^−1^. HRMS: *m*/*z* calcd. for C_15_H_22_O_5_ ([M+H]^+^): 283.1521, found: 283.1540; calcd. for ([M+Na]^+^): 305.1360, found: 305.1359. Elemental Analysis calcd. for C_15_H_22_O_5_: C: 63.81%, H: 7.85%, found: C: 63.82%, H: 7.90%.

*Crystal Data for **4a***: C_15_H_22_O_5_, M = 282.32 g·mol^−1^, monoclinic, *I*2/*a*, λ = 1.54184 Å, *a* = 18.1382 (3) Å, *b* = 11.3690 (2) Å, *c* = 15.9193 (4) Å, β = 118.261 (2) °, *V* = 2891.44(12) Å^3^, *Z* = 8, ρ_calcd_ = 1.297 Mg∙m^−3^, μ = 0.798 mm^−1^, T = 110 (2) K, *θ* range 3.10°–61.986°, 4314 reflections collected, 2243 independent reflections (*R*_int_ = 0.0233), R1 = 0.0452, *w*R2 = 0.1172 (I > 2σ(I)).

### 3.6. Synthesis of Methyl d4-(E)-2-(2,2-Dimethoxy-4,5,6,7,8,9-hexahydrocycloocta[b]furan-3(2H)-ylidene)acetate *(**4b**)*

As described for **4a**, cyclooctyne was analogously treated with CD_3_OD and **1a**, and workup led to **4b** (447 mg, 62%) as a colorless solid with m.p. 63–64 °C (from cyclohexane/*tert*-butyl methyl ether). ^1^H-NMR (CDCl_3_): δ = 1.35–1.42 (m, 2H, 6'-CH_2_), 1.46–1.53 (m, 2H, 7'-CH_2_), 1.57–1.65 (m, 2H, 5'-CH_2_), 1.64–1.71 (m , 2H, 8'-CH_2_), 2.42 (m, 2H, 9'-CH_2_), 2.69 (t, *J* = 6.3, 2H, 4'-CH_2_), 3.27 (s, 3H, 2'-OCH_3_), 3.63 (s, 3H, CO_2_CH_3_). ^2^H-NMR (CHCl_3_:CDCl_3_ = 9:1): δ = 3.30 (s, 3D, 2'-OCD_3_), 5.53 (s, 1D, 2-CD). ^13^C-NMR (CDCl_3_): δ = 22.0 (t, C-4'), 25.5 (t, C-6'), 26.4 (t, C-7'), 26.9 (t, C-9'), 28.6 (t, C-8'), 29.2 (t, C-5'), 50.4 (s (DEPT), sept with *^1^J*_CD_ = 22.0, 2'-OCD_3_), 50.9 (q, OCH_3_), 51.1 (q, OCH_3_), 105.8 (s (DEPT), t with *^1^J*_CD_ = 24.5, C-2), 112.4 (s, C-3a'), 121.6 (s, C-2'), 152.0 (s, C-3'), 166.1 (s, C-1), 170.2 (s, C-9a'). HRMS: *m*/*z* calcd. for C_15_H_18_D_4_O_5_ ([M+H]^+^): 287.1791, found: 287.1772; calcd. for ([M+Na]^+^): 309.1611, found: 309.1633; calcd. for ([M+K]^+^): 325.1350, found: 325.1367.

### 3.7. Synthesis of Methyl (E)-2-(2-methoxy-2-phenoxy-4,5,6,7,8,9-hexahydrocycloocta[b]furan-3(2H)-ylidene)acetate *(**4c**)*

To a solution of phenol (3.00 g, 31.9 mmol) and cyclooctyne (0.40 g, 3.7 mmol) in anhydrous THF (4 mL), **1a** (1.05 g, 7.4 mmol) was added with the help of a syringe within 2 h. Thereafter, the mixture, which changed its color to deep red, was stirred at ambient temperature for 16 h. After removal of the solvent at reduced pressure, the residue was dissolved in Et_2_O (40 mL) and washed with aqueous sodium hydroxide (10%, 2 L). After drying of the organic phase (MgSO_4_) and removal of the solvent, the residue was purified by chromatography (first SiO_2_, CH_2_Cl_2_, then basic Al_2_O_3_, CH_2_Cl_2_) to furnish a green oil that was treated with hexane. This led to the precipitation of a colorless solid, which was removed by filtration. After removal of the solvent, **4c** (247 mg, 10%) was obtained as a green oil that can be further purified by HPLC (LiChrospher Si 60 (5μ), 20 × 2 cm, CH_2_Cl_2_, 20 mL/min). ^1^H-NMR (CDCl_3_, relaxation delay d_1_ = 15 s): δ = 1.00–1.72 (m, 8H), 2.38–2.41 (m, 2H), 2.53–2.60 (m, 1H), 2.77–2.83 (m, 1H), 3.44 (s, 3H, 2'-OCH_3_), 3.70 (s, 3H, CO_2_CH_3_), 5.74 (s, 1H, 2-H), 7.06–7.25 (m, 5H, O-Ph). ^13^C-NMR (CDCl_3_): δ = 22.0 (t, C-4'), 25.1 (t, C-6'), 26.2 (t, C-7'), 26.9 (t, C-9'), 28.3 (t, C-8'), 29.1 (t, C-5'), 51.1 (q, 1-OCH_3_), 51.5 (q, 2'-OCH_3_), 107.5 (d, C-2), 112.7 (s, C-3a'), 121.1 (d, Ph), 122.3 (s, C-2'), 124.4 (d, Ph), 128.8 (d, Ph), 152.3 (s, C-3'), 152.4 (s, Ph), 166.2 (s, C-1), 169.7 (s, C-9a'). IR (CDCl_3_): = 2931 (s), 1705 (s, C=O) 1506 (s), 1490 (s) cm^−1^.

### 3.8. Synthesis of Dimethyl (E)-2-(2-oxocyclooctylidene)succinate [(E)-5] and Dimethyl (Z)-2-(2-oxocyclooctylidene)succinate [(Z)-**5**]

To THF (2 mL) and water (1 mL), cyclooctyne (0.41 g, 3.84 mmol) and **1a** (0.56 g, 3.94 mmol) were added with stirring at 0 °C. Thereafter, the mixture was stirred at ambient temperature for 21 h. This led to the formation of an inhomogeneous mixture, which was diluted with diethyl ether (50 mL) and dried (MgSO_4_). After removal of the solvent under reduced pressure, the resulting yellow liquid was analyzed by ^1^H-NMR, which indicated a 5:1 ratio of (*E*)-**5** and (*Z*)-**5**. By using flash chromatography (SiO_2_, Et_2_O/hexane 1:2), (*E*)-**5** (0.22 g, 21%) and (*Z*)-**5** (0.20 g, 20%) were isolated as colorless liquids.

(*E*)-**5**: ^1^H-NMR (CDCl_3_): δ = 1.42‒1.48 (m, 2H), 1.52‒1.58 (m, 2H), 1.61‒1.68 (m, 2H), 1.78‒1.86 (m, 2H), 2.46‒2.51 (m, 2H, 8'-H), 2.72‒2.76 (m, 2H, 3'-H), 3.25 (s, 2H, 3-H), 3.64 (s, 3H, OCH_3_), 3.74 (s, 3H, OCH_3_). ^13^C-NMR (CDCl_3_): δ = 23.64 (t, CH_2_), 25.51 (t, CH_2_), 25.77 (t, CH_2_), 27.56 (t, CH_2_), 32.44 (t, C-8'), 36.04 (t, C-3), 43.46 (t, C-3'), 52.03 (q), 52.14 (q), 122.34 (s), 154.13 (s), 167.33 (s), 170.79 (s), 212.43 (s, C-2'). IR (CCl_4_): = 1745 (s) cm^−1^. Elemental Analysis calcd. for C_14_H_20_O_5_: C: 62.67%, H: 7.51%. found: C: 62.37%, H: 7.46%.

(*Z*)-**5**: ^1^H-NMR (CDCl_3_): δ = 1.44–1.63 (m, 6H), 1.75‒1.82 (m, 2H), 2.40‒2.44 (m, 2H, 8'-H), 2.62‒2.66 (m, 2H, 3'-H) 3.38 (s, 2H, 3-H), 3.68 (s, 6H, 2 × OCH_3_). ^13^C-NMR (CDCl_3_): δ = 23.62 (t, CH_2_), 25.49 (t, CH_2_), 25.74 (t, CH_2_), 27.53 (t, CH_2_), 32.40 (t, C-8'), 36.00 (t, C-3), 43.43 (t, C-3'), 51.99 (q, OCH_3_), 52.09 (q, OCH_3_), 122.32 (s), 154.08 (s), 167.28 (s), 170.74 (s), 212.36 (s, C-2'). IR (CCl_4_): = 1745 (s) cm^‒1^. Elemental Analysis calcd. for C_14_H_20_O_5_: C: 62.67%, H: 7.51%, found: C: 62.43%, H: 7.36%.

### 3.9. Synthesis of Dimethyl 2-Methoxy-1-[(2-methoxycarbonyl)-3-methyloxiran-2-yl]-5,6,7,8,9,10-hexahydro-2H-2,4a-epoxybenzo[8]annulene-3,4-dicarboxylate *(**6a**)*

To a solution of cyclooctyne (410 mg, 3.79 mmol) in acetaldehyde (3 mL, freshly distilled), a solution of **1a** (540 mg, 3.79 mmol) in anhydrous THF (2 mL) was added with the help of a syringe within 2 h. The yellow mixture was stirred at room temperature overnight. Thereafter, the solvent and the excess of acetaldehyde were removed at reduced pressure, and the resulting yellow oil was purified by flash chromatography (SiO_2_, Et_2_O/hexane 1:1). The oily product was recrystallized twice from Et_2_O/hexane (1:3) to give **6a** (180 mg, 22%) as colorless crystals with m.p. 66–67 °C. Single crystals, which were appropriate for X-ray diffraction analysis, were obtained by slow evaporation of a solution of **6a** in cyclohexane. ^1^H-NMR (CDCl_3_): δ = 1.38 (d, *^3^J* = 5.6, 3H, C–CH_3_), 1.25–1.91 (m, 8H), 2.11 (m, 2H, 5-H), 2.51 (ddd, *^3^J*_10-H(a),9-H(a)_ = 15, *^2^J*_10-H(a),10-H(b)_ = 13.2, *^3^J*_10-H(a),9-H(b)_ = 4.8, 1H, 10-H(a)), 2.66 (dt, *^2^J*_10-H(a),10-H(b)_ = 13.2, *^3^J*_10-H(b),9-H(a)_ = 4, *^3^J*_10-H(b),9-H(b)_ = 4, 1H, 10-H(b)), 3.61 (s, 3H, OCH_3_), 3.68 (q, *^3^J*_3'-H,CH3_ = 5.6, 1 H, 3'-H), 3.73 (s, 3H, OCH_3_), 3.779 (s, 3H, OCH_3_), 3.783 (s, 3H, OCH_3_). ^13^C-NMR (CDCl_3_): δ = 13.74 (q, CH_3_), 23.01 (t, CH_2_), 23.21 (t, CH_2_), 23.37 (t, CH_2_), 25.13 (t, CH_2_), 25.58 (t, CH_2_), 29.60 (t, CH_2_), 52.24 (q, 2 OCH_3_), 52.54 (q, OCH_3_), 55.01 (q, OCH_3_), 57.60 (s, C-2'), 57.86 (d, C-3'), 89.53 (s, C-4a), 115.48 (s, C-2), 139.33 (s), 150.29 (s), 156.52 (s), 163.22 (s), 163.91 (s), 164.18 (s), 168.35 (s). IR (CCl_4_): = 1709 (s) cm^−1^. Elemental Analysis calcd. for C_22_H_28_O_9_: C: 60.54%, H: 6.47%. found: C: 60.55%, H: 6.45%.

*Crystal Data for* **6a**: C_22_H_28_O_9_, M = 436.44 g∙mol^−1^, monoclinic, *P*2_1_/*c*, λ = 1.54184 Å, *a* = 8.8577 (4) Å, *b* = 28.5456 (16) Å, *c* = 17.3589 (9) Å, β = 90.063 (4) °, *V* = 4389.2 (4) Å^3^, *Z* = 8, ρ_calcd_ = 1.321 Mg∙m^−3^, μ = 0.863 mm^−1^, T = 100 (2) K, *θ* range 3.10°−61.77°, 28823 reflections collected, 6811 independent reflections (*R*_int_ = 0.0557), R1 = 0.0548, *w*R2 = 0.1478 (I > 2σ (I)).

### 3.10. Synthesis of Dimethyl 2-Methoxy-1-[(2-methoxycarbonyl)-3-phenyloxiran-2-yl]-5,6,7,8,9,10-hexahydro-2H-2,4a-epoxybenzo[8]annulene-3,4-dicarboxylate *(**6b**)*

To a solution of cyclooctyne (270 mg, 2.53 mmol) in benzaldehyde (4 mL, freshly distilled), a solution of **1a** (1.10 g, 7.58 mmol) in anhydrous THF (2 mL) was added with the help of a syringe within 2 h. The orange mixture was stirred overnight at room temperature. Thereafter, the solvent was evaporated and the excess of benzaldehyde was removed at 50 °C and 9 × 10^−2^ mbar. The resulting yellow oil was purified by flash chromatography (SiO_2_, Et_2_O/hexane 1:1). The oily product was crystallized from Et_2_O/hexane (ratio 1:3) to give **6b** (260 mg, 21%) as colorless crystals. ^1^H NMR data indicated that the substance consisted of two diastereomers in a 4:1 ratio. IR (mixture of isomers): *=* 1711 (s) cm^−1^. Elemental Analysis (mixture of isomers) calcd. for C_27_H_30_O_9_: C: 65.05%, H: 6.07%, found: C: 64.77%, H: 6.02%. The diastereomers of **6b** were (partly) separated by flash chromatography (SiO_2_, CH_2_Cl_2_); the minor isomer was eluted before the main isomer.

Minor isomer: ^1^H-NMR (CDCl_3_): δ = 1.25–2.10 (m, 8H), 2.18 (m, 2H, 5-H), 2.54 (ddd, *^3^J*_10-H(a),9-H(a)_ = 15.2, *^2^J*_10-H(a),10-H(b)_ = 13, *^3^J*_10-H(a),9-H(b)_ = 4.8, 1H, 10-H(a)), 2.72 (dt, *^2^J*_10-H(a),10-H(b)_ = 13, *^3^J*_10-H(b), 9-H(a)_ = 4, *^3^J*_10-H(b),9-H(b)_ = 4, 1H, 10-H(b)), 3.39 (s, 3H, OCH_3_), 3.75 (s, 3H), 3.779 (s, 3H), 3.783 (s, 3H), 4.32 (s, 1H, 3'-H), 7.28–7.41 (m, 5 H, Ph). ^13^C-NMR (CDCl_3_): δ = 23.05 (t, CH_2_), 23.44 (t, CH_2_), 23.69 (t, CH_2_), 25.37 (t, CH_2_), 25.77 (t, CH_2_), 30.11 (t, CH_2_), 52.08 (q, CH_3_), 52.24 (q, 2C), 55.40 (q, CH_3_), 60.66 (s, C-2'), 63.11 (d, C-3'), 89.18 (s, C-4a), 115.82 (s, C-2), 126.51 (d, 2C), 128.00 (d, 2C), 128.29 (d, CH), 133.07 (s), 139.82 (s), 150.77 (s), 157.34 (s), 162.24 (s), 163.01 (s), 164.35 (s), 166.51 (s).

Major isomer: M.p. 116–118 °C. ^1^H-NMR (CDCl_3_): δ = 1.31–2.22 (m, 10 H), 2.55 (ddd, *^3^J*_10-H(a),9-H(a)_ = 15.2, *^2^J*_10-H(a),10-H(b)_ = 13, *^3^J*_10-H(a),9-H(b)_ = 4.8, 1 H, 10-H(a)), 2.85 (dt, *^2^J*_10-H(a),10-H(b)_ = 13, *^3^J*_10-H(b),9-H(a)_ = 4, *^3^J*_10-H(b),9-H(b)_ = 4, 1H, 10-H(b)), 3.42 (s, 3H, OCH_3_), 3.70 (s, 3H), 3.79 (s, 6H), 4.71 (s, 1H, 3'-H), 7.28–7.41 (m, 5H, Ph). ^13^C-NMR (CDCl_3_): δ = 22.94 (t, CH_2_), 23.20 (t, CH_2_), 23.45 (t, CH_2_), 25.08 (t, CH_2_), 25.72 (t, CH_2_), 29.82 (t, CH_2_), 52.16 (q, CH_3_), 52.28 (q, 2C), 55.10 (q, CH_3_), 60.54 (s, C-2'), 61.89 (d, C-3'), 89.67 (s, C-4a), 115.54 (s, C-2), 126.28 (d, 2C), 128.06 (d, 2C), 128.34 (d, CH), 133.25 (s), 138.99 (s), 150.43 (s), 156.48 (s), 163.21 (s), 163.80 (s), 165.26 (s), 166.65 (s).

### 3.11. Synthesis of (Z)-Trimethylsilyl 2-(2-Oxo-4,5,6,7,8,9-hexahydrocycloocta[b]furan-3(2H)-ylidene)-2-(trimethylsilyl)acetate *(**7**)*

To a solution of bis(trimethylsilyl) but-2-ynedioate (0.10 g, 0.37 mmol) in anhydrous methylene chloride (2 mL), cyclooctyne (0.06 g, 0.55 mmol) was added under nitrogen atmosphere. The mixture was stirred for 48 h at 40 °C. The solvent and the excess of cyclooctyne were evaporated under reduced pressure to give 0.17 g (85%) of an unstable yellow oil. ^1^H-NMR (CDCl_3_): δ = 0.23 (s, 9H, CSi(CH_3_)_3_), 0.30 (s, 9H, CO_2_Si(CH_3_)_3_), 1.44–1.60 (m, 6H, CH_2_), 1.69–1.75 (m, 2 H, CH_2_), 2.31 (t, *J* = 6.4, 2 H, CH_2_), 2.48 (t, *J* = 7.1, 2 H, CH_2_). ^13^C-NMR(CDCl_3_): *δ* = −1.48 (q, CSi(*C*H_3_)_3_), −0.23 (q, OSi(CH_3_)_3_), 21.11 (t, CH_2_), 25.30 (t, CH_2_), 25.85 (t, CH_2_), 26.00 (t, CH_2_), 27.02 (t, CH_2_), 28.15 (t, CH_2_), 114.48 (s, C-3a'), 133.46 (s, C=*C*-COOC), 146.84 (s, *C*=CCOOC), 158.85 (s, C-9a'), 168.35 (s, CO), 171.09 (s, CO). Signal assignment and determination of the stereochemistry were supported by HMBC and NOESY experiments, respectively. ^29^Si-NMR (CDCl_3_): δ = −3.86 (C*Si*(CH_3_)_3_), 25.59 (O*Si*(CH_3_)_3_). IR (CCl_4_): = 3010 (w), 2933 (s), 1785 (s), 1688 (s), 1249 (s) cm^−1^. HRMS: *m*/*z* calcd. for: C_18_H_30_O_4_Si_2_ ([M+Na]^+^): 389.1580, found: 389.1643.

## 4. Conclusions

In summary, it was shown in this study that cyclooctynes and acetylenedicarboxylates react under mild conditions to generate short-lived dipolar intermediates of type **2** via an unusual 1,3-dipolar cycloaddition. These intermediates can be trapped by molecules with electron-deficient π-systems to give complex polycyclic products through a cascade of cycloaddition reactions. On the other hand, interception of **2** in the presence of protic reaction partners leads to 2,3-dihydrofuran derivatives with the rare substitution pattern of an orthoester functionality and a 3-methylidene group [36,37,38]. In all cases, the products are formed with perfect atom economy, that means that all atoms of the substrate molecules are found in the product.

Currently, we investigate scope and limitations of the presented method to prepare furan derivatives and whether acetylenedicarboxylates can be substituted by prop-2-ynoic esters with different electron-withdrawing substituents in 3-position or by the analogous amides to generate intermediates similar to **2** and the corresponding subsequent products.

## Supplementary Materials

Supplementary materials can be accessed at: www.mdpi.com/1420-3049/19/9/14022/s1.

CCDC 1016789 (**3b**), 1016790 (**4a**) and 1016788 (**6a**) contains the supplementary crystallographic data for this paper. These data can be obtained free of charge via http://www.ccdc.cam.ac.uk/conts/retrieving.html (or from the CCDC, 12 Union Road, Cambridge CB2 1EZ, UK; Fax: +44 1223 336033; E-mail: deposit@ccdc.cam.ac.uk).

## Supplementary Files

Supplementary File 1
