# Supplementary material for: Synthesis with Perfect Atom Economy: Generation of Furan Derivatives by 1,3-Dipolar Cycloaddition of Acetylenedicarboxylates at Cyclooctynes
Source: Molecules. 2014 Sep 5;19(9):14022–35. doi: 10.3390/molecules190914022 (PMC6271402; doi:10.3390/molecules190914022)

Supplementary Materials

Figure S1. ^{1}H NMR spectrum of **3b**.

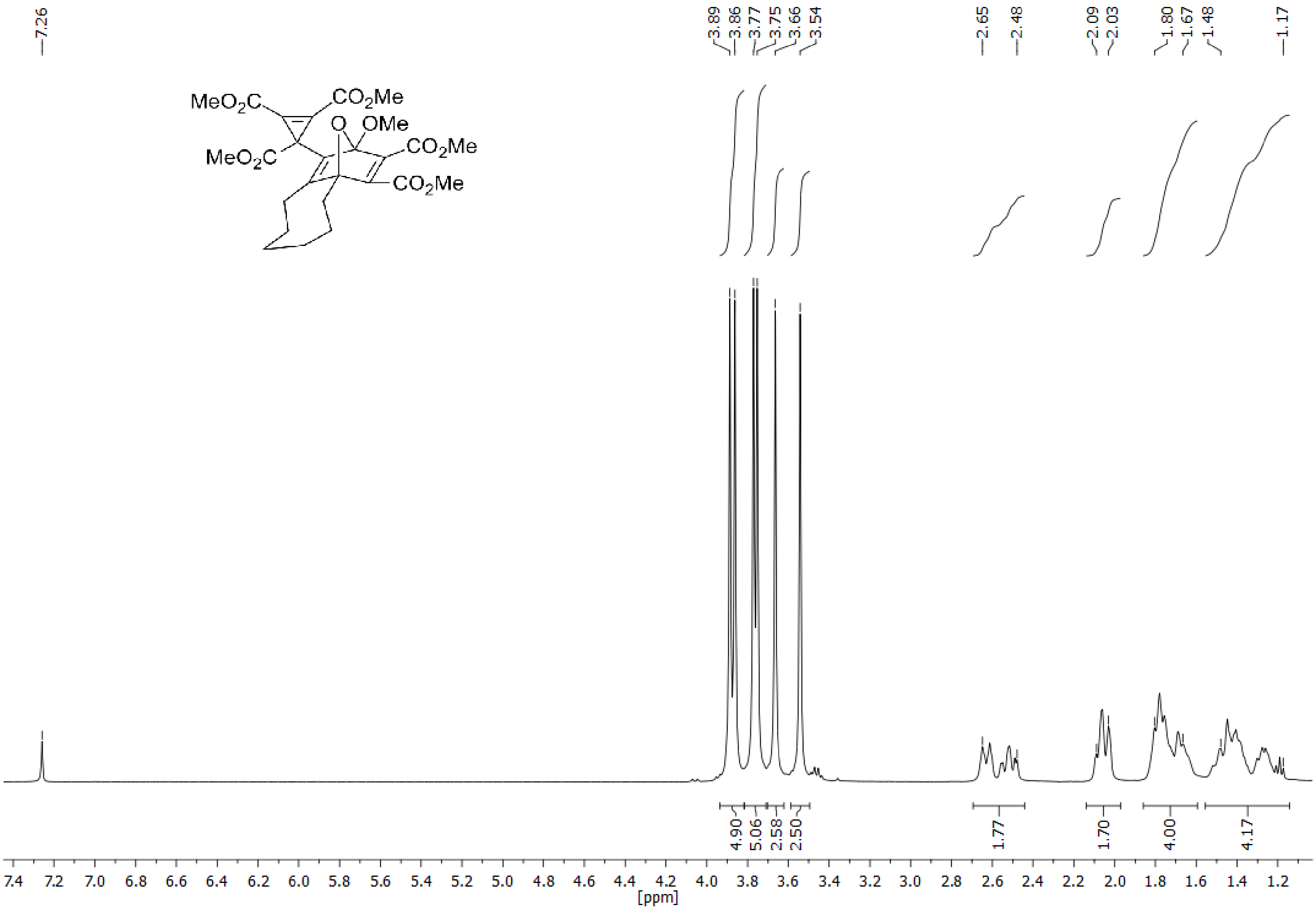

Figure S2. ^{13}C NMR spectrum of **3b**.

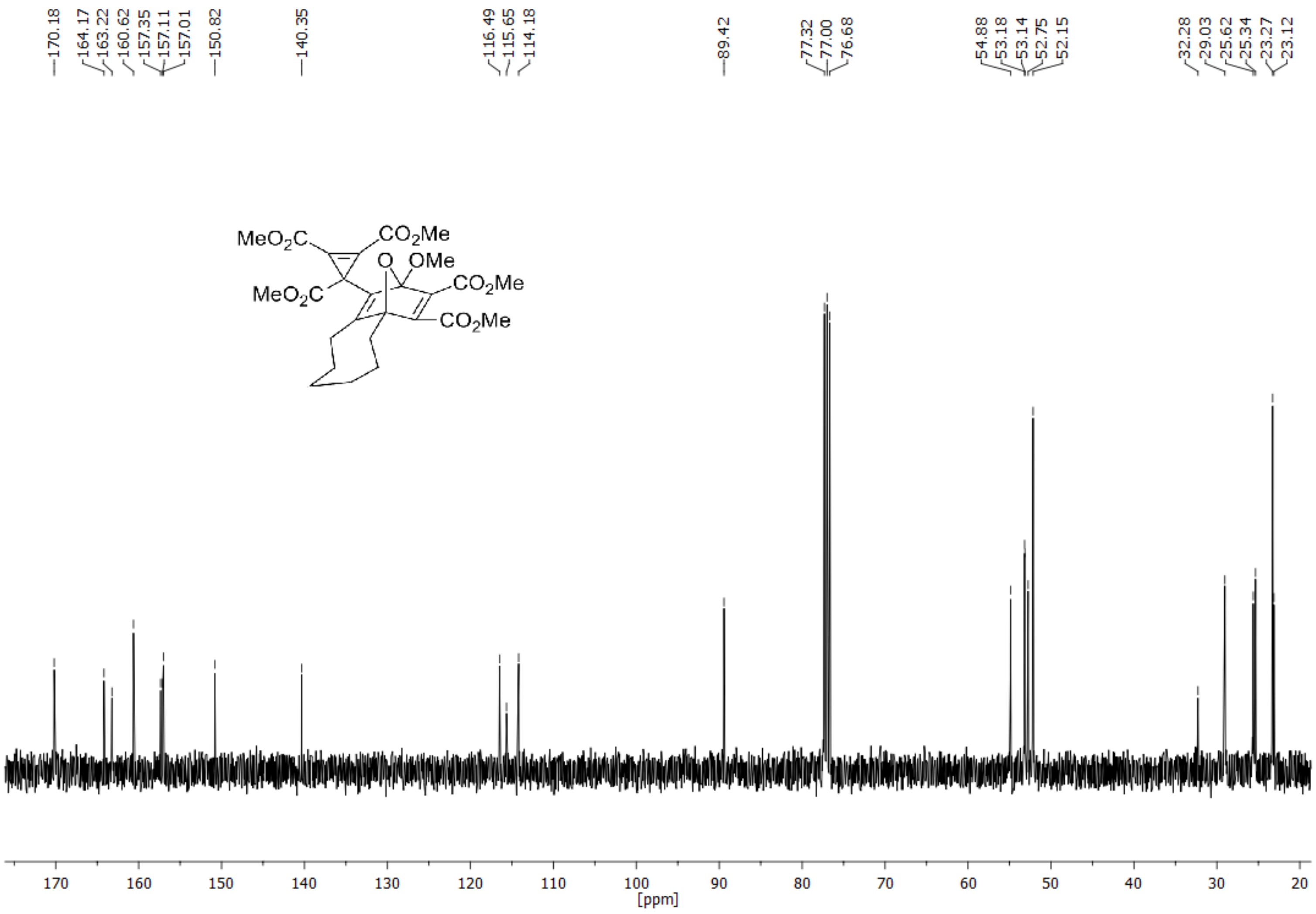

Figure S3. ^{1}H-NMR spectrum of **3c**.

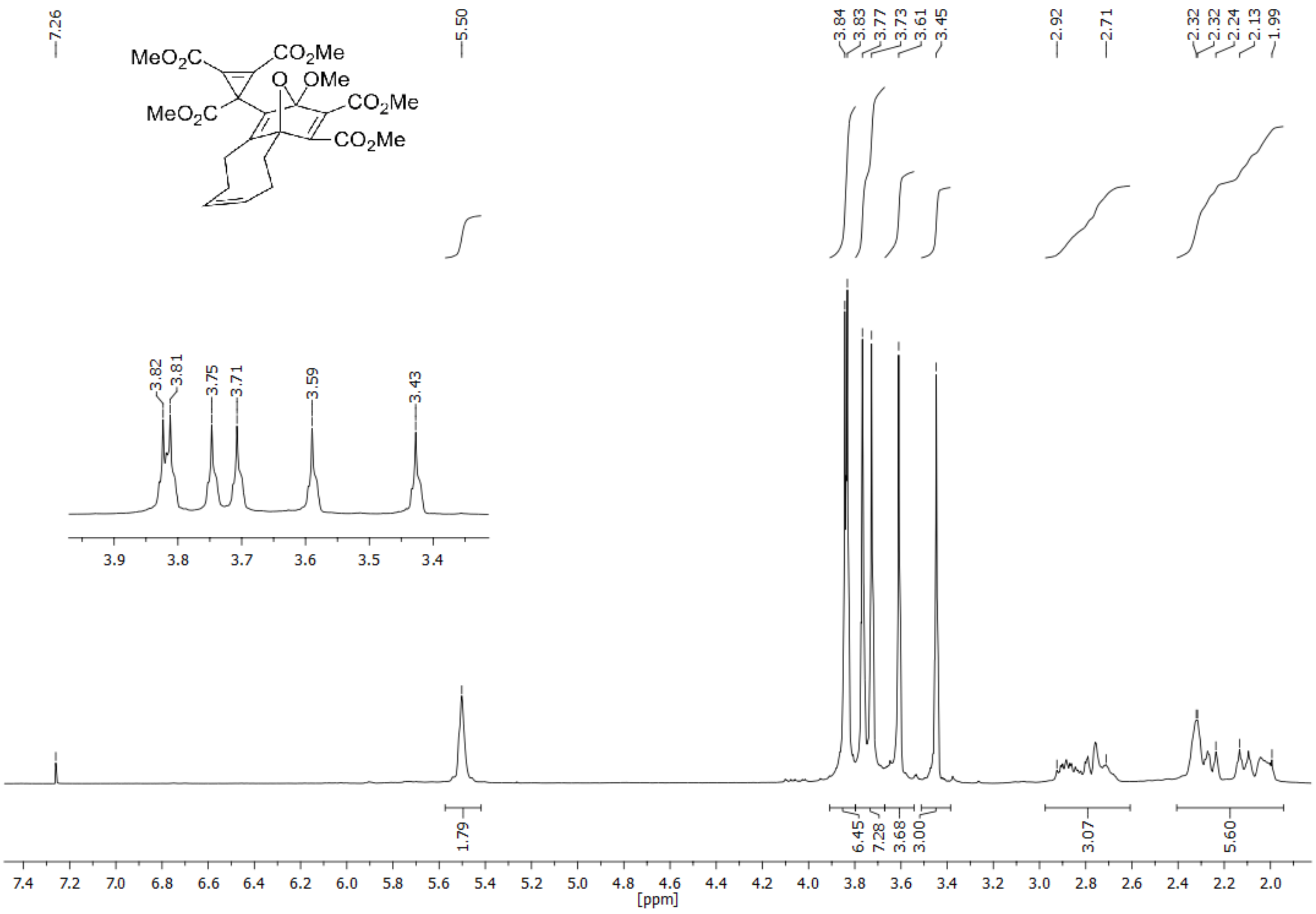

Figure S4. ^{13}C NMR spectrum of **3c**.

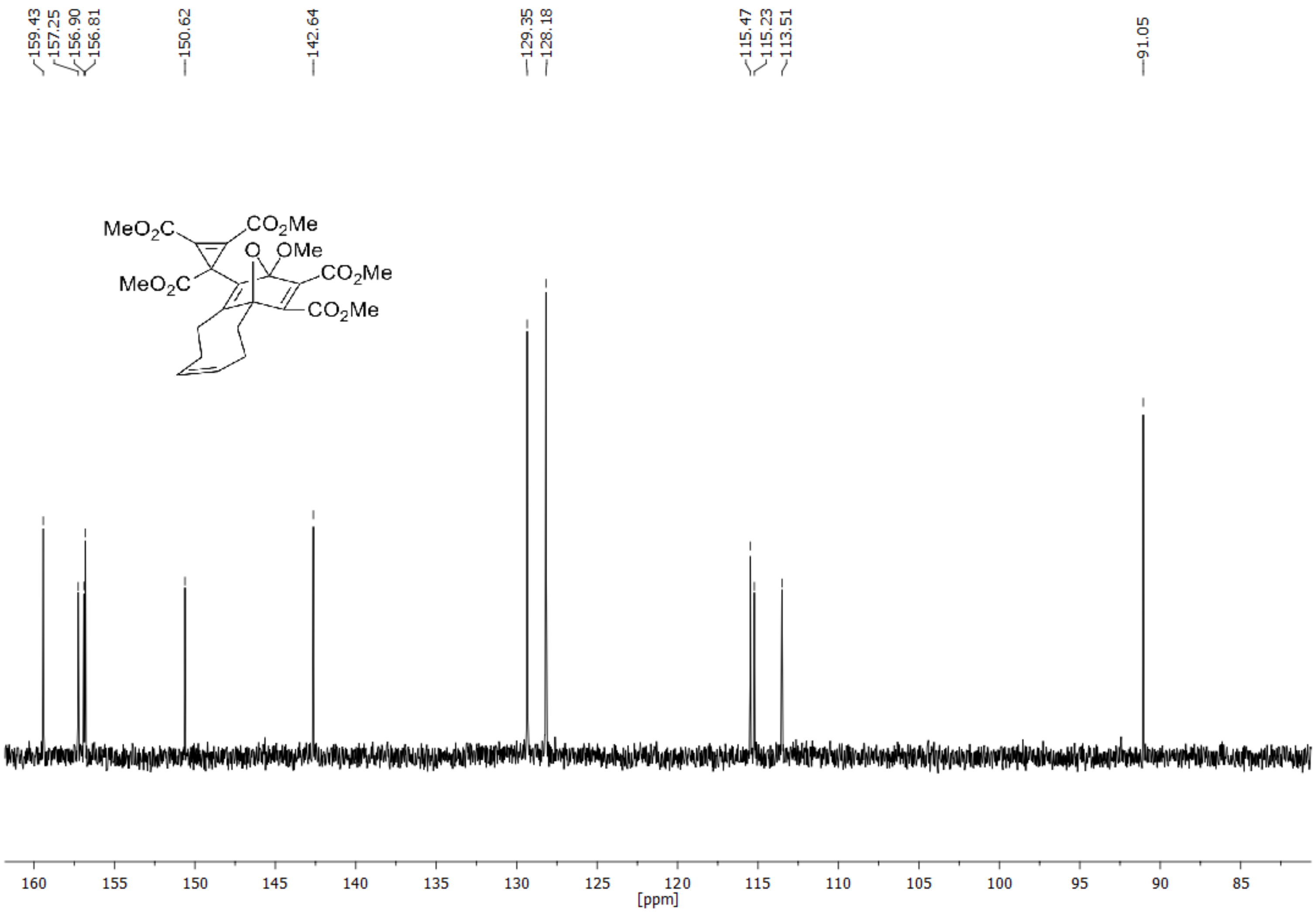

Figure S5. ^{1}H NMR spectrum of **3d**.

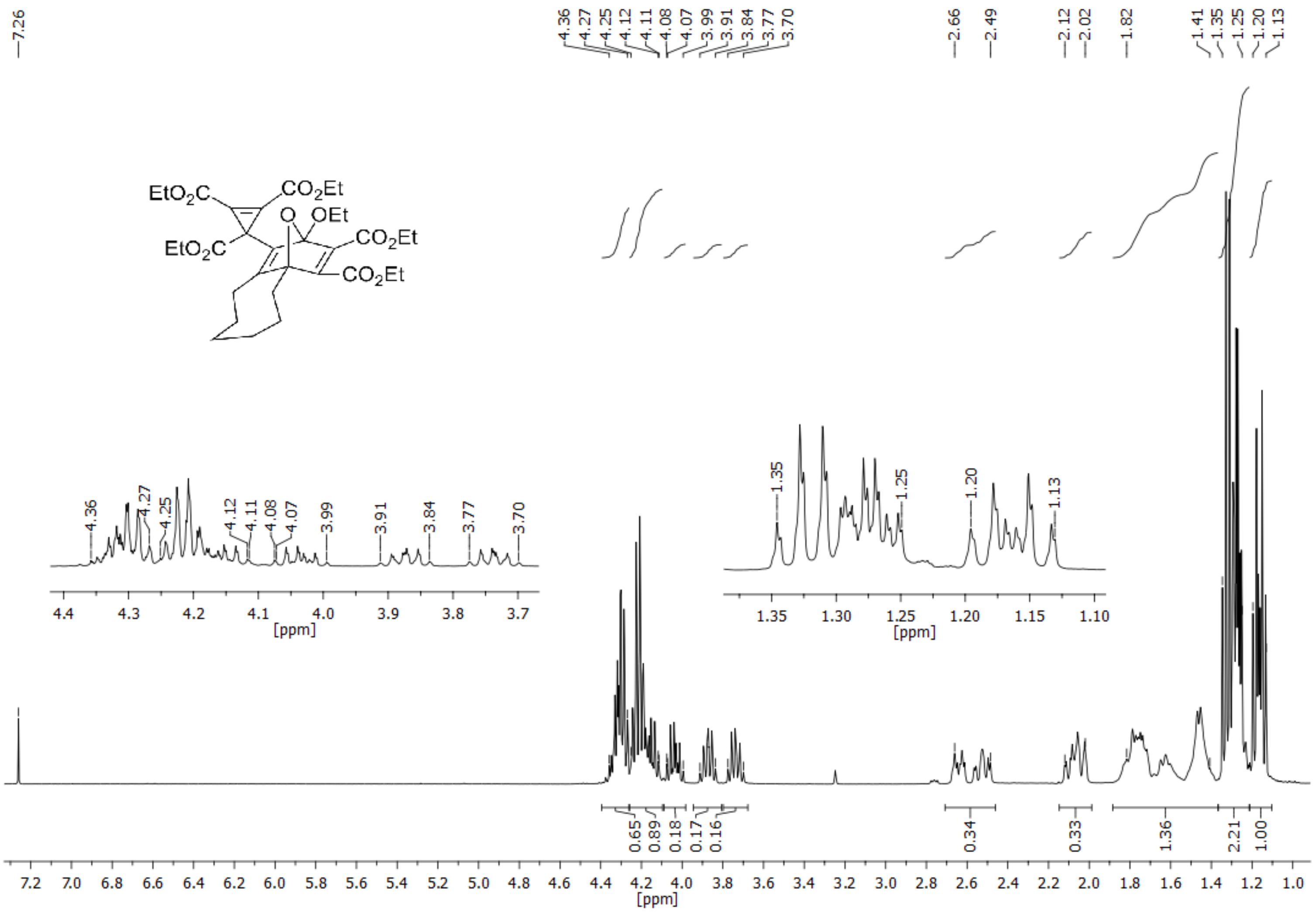

Figure S6. ^{13}C NMR spectrum of **3d**.

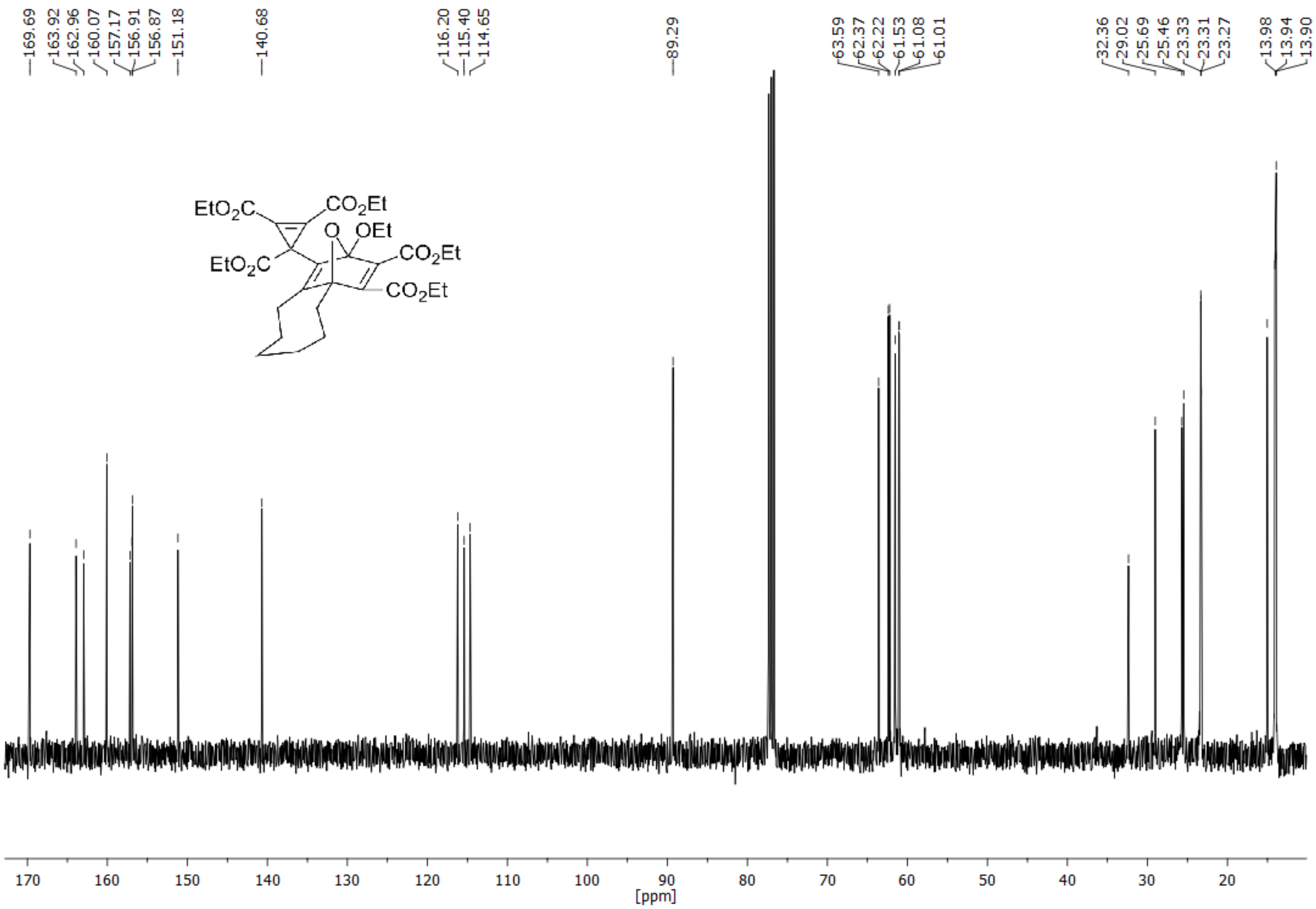

Figure S7. ^{1}H NMR spectrum of **4a**.

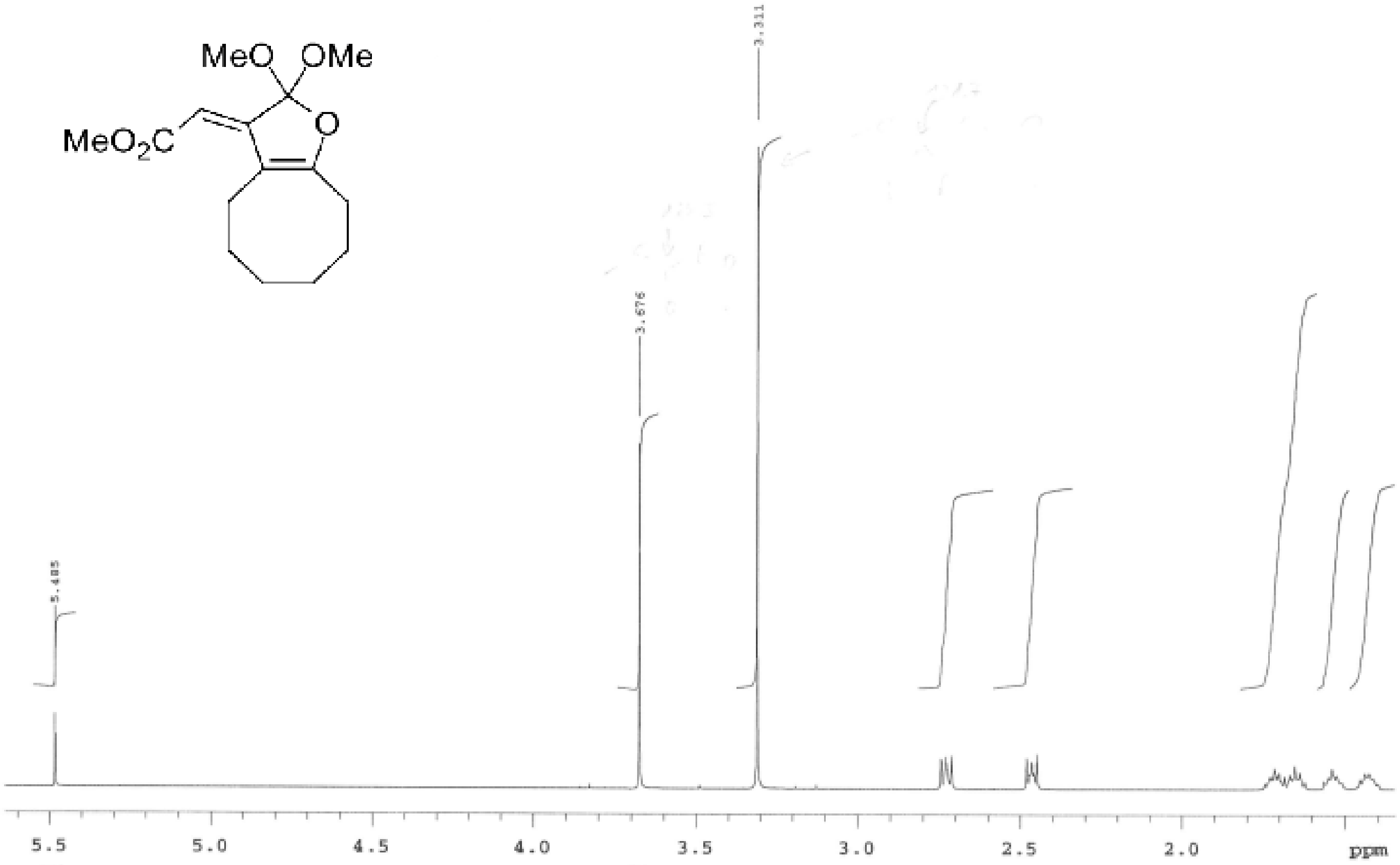

Figure S8. ^{13}CNMR spectrum of **4a**.

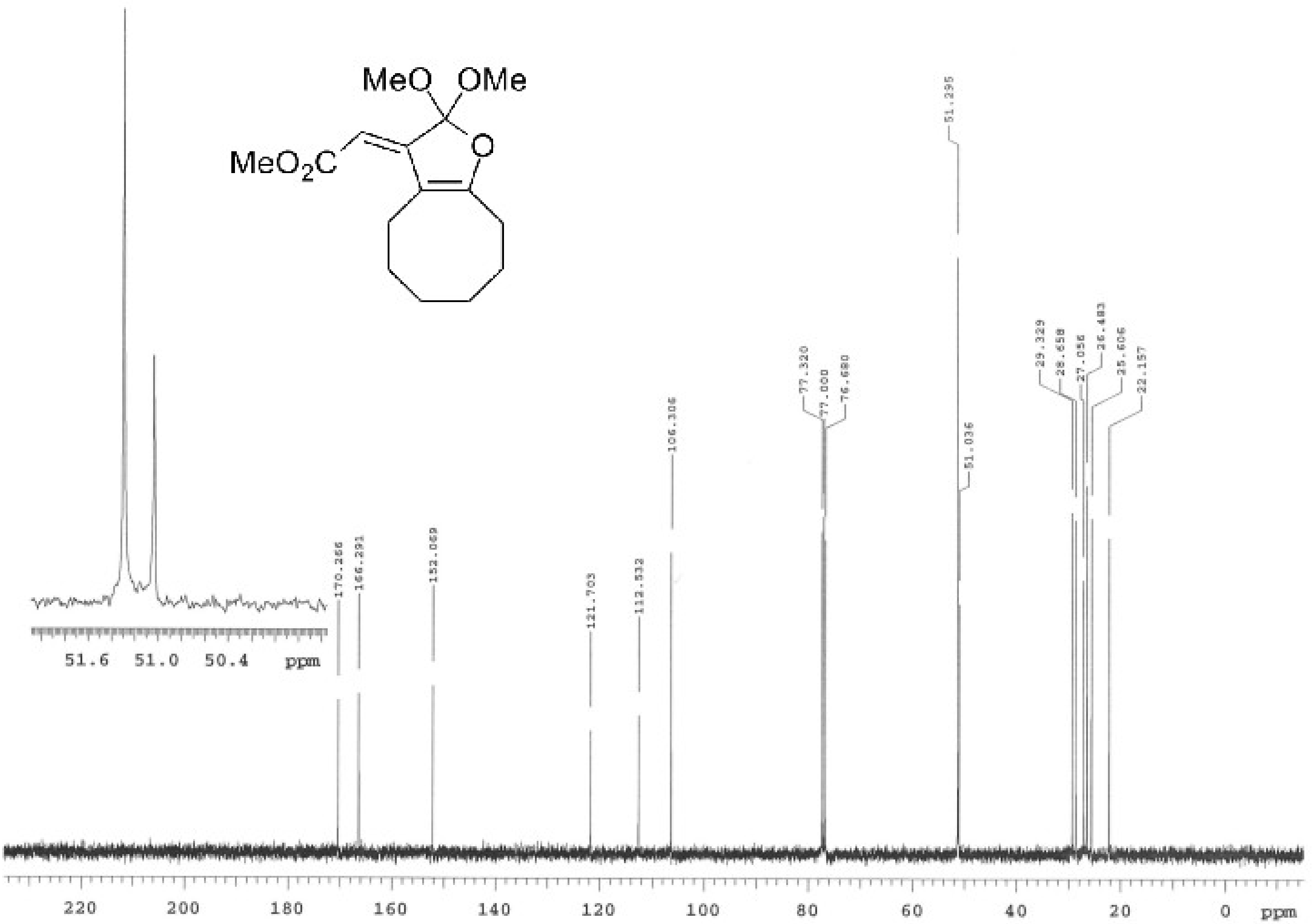

Figure S9. IR spectrum of **4a**.

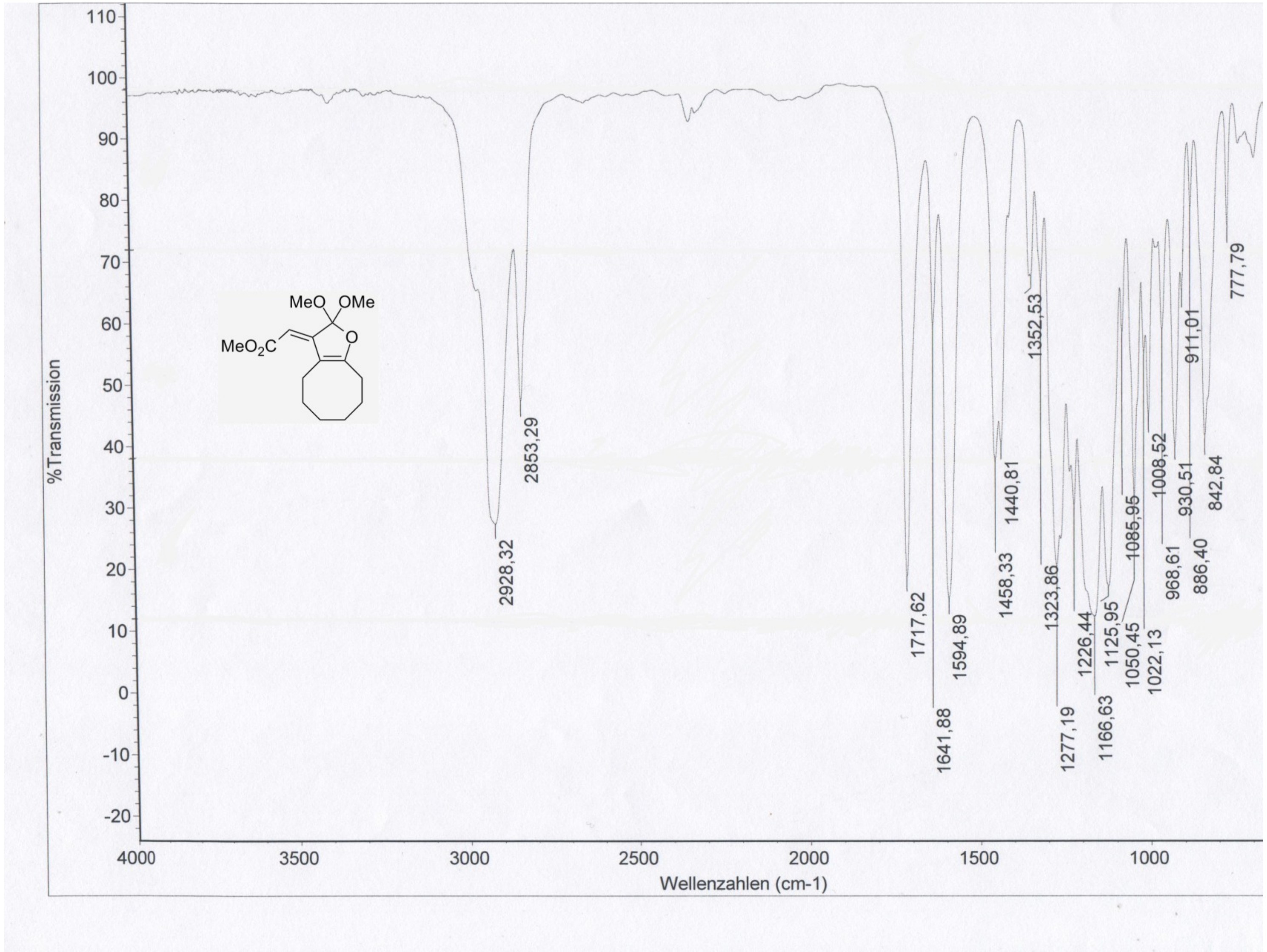

Figure S10. ^{1}H NMR spectrum of **4b**.

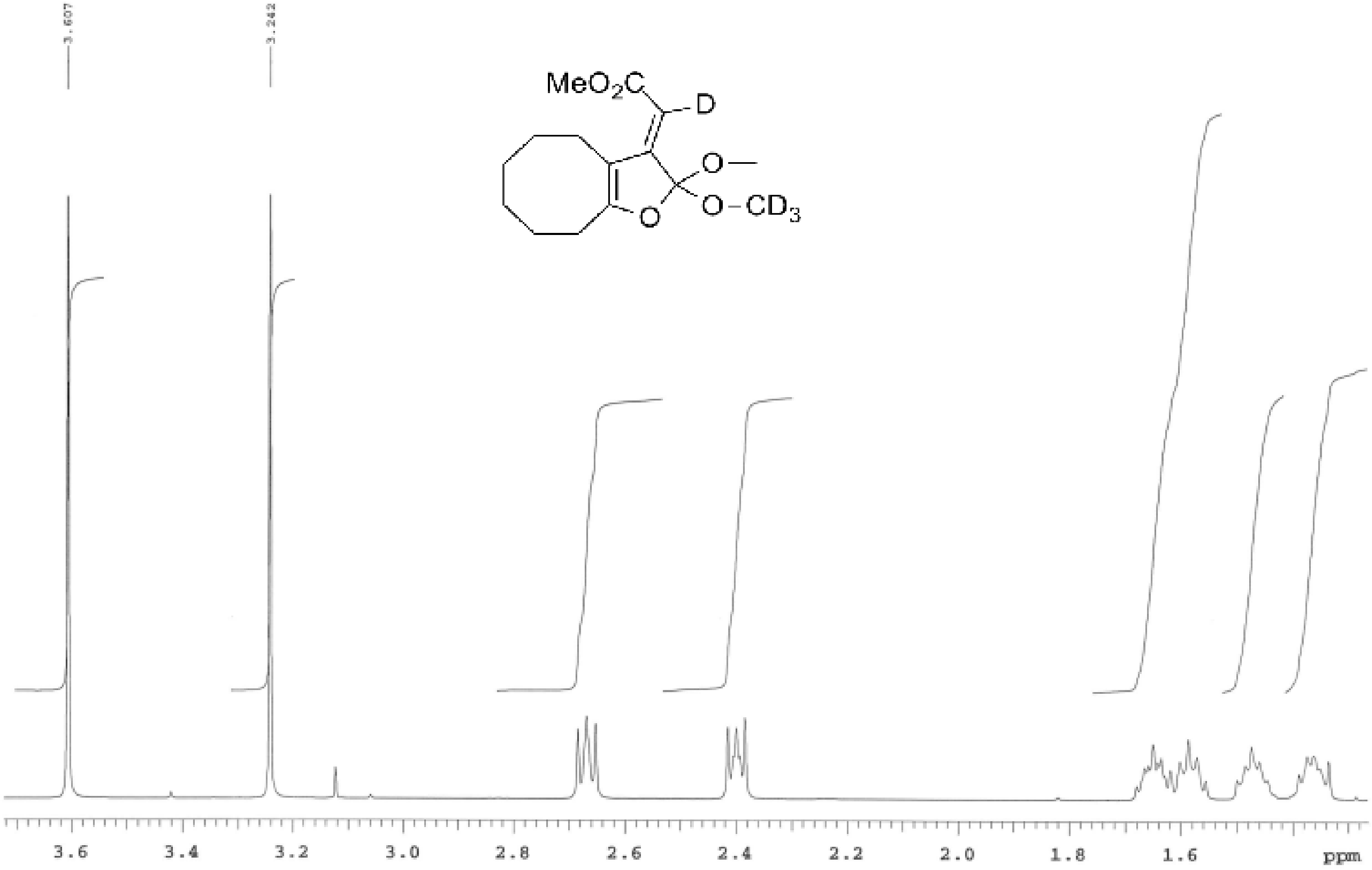

Figure S11. ^{13}C NMR spectrum of **4b**.

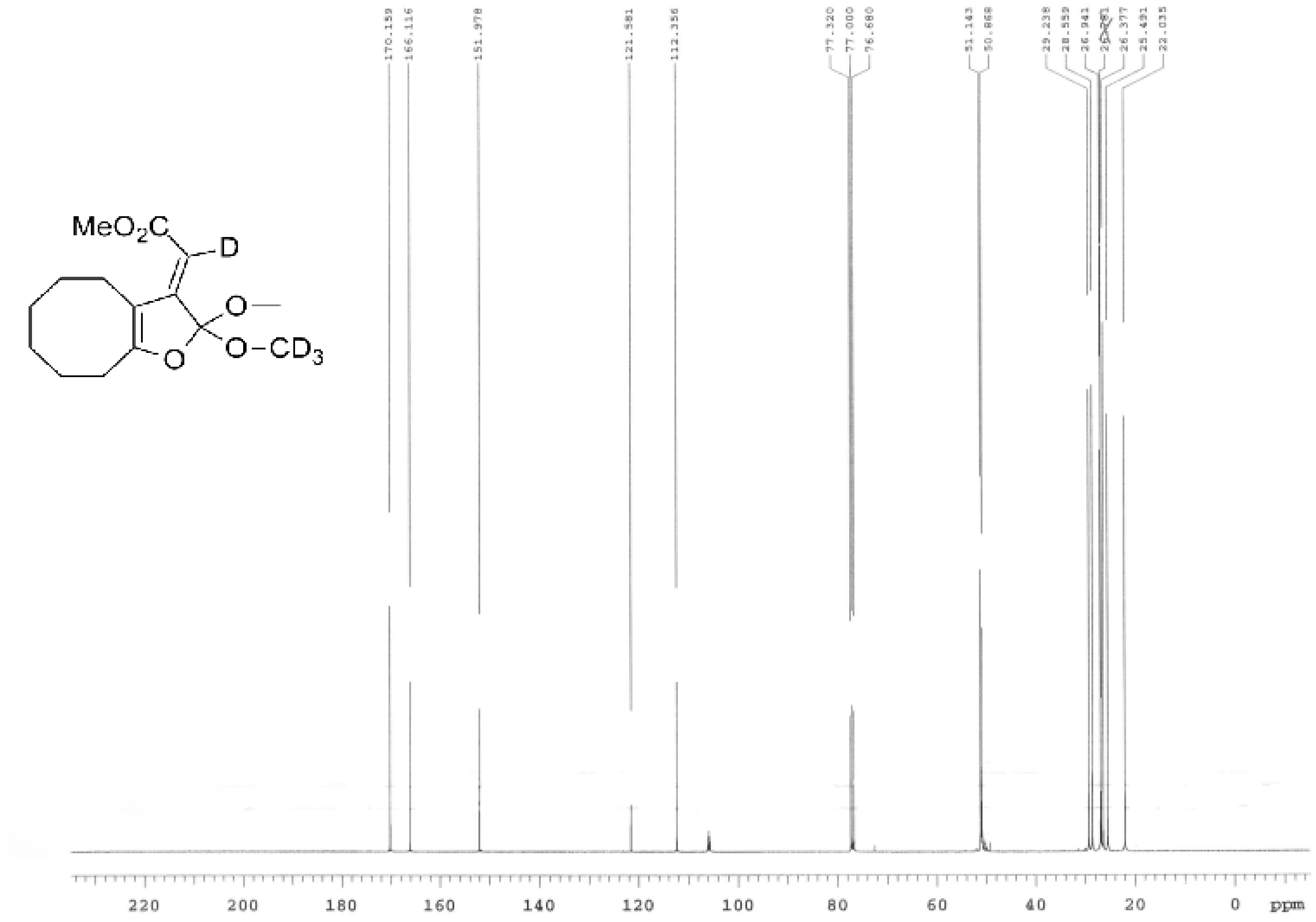

Figure S12. ^{1}H NMR spectrum of **4c**.

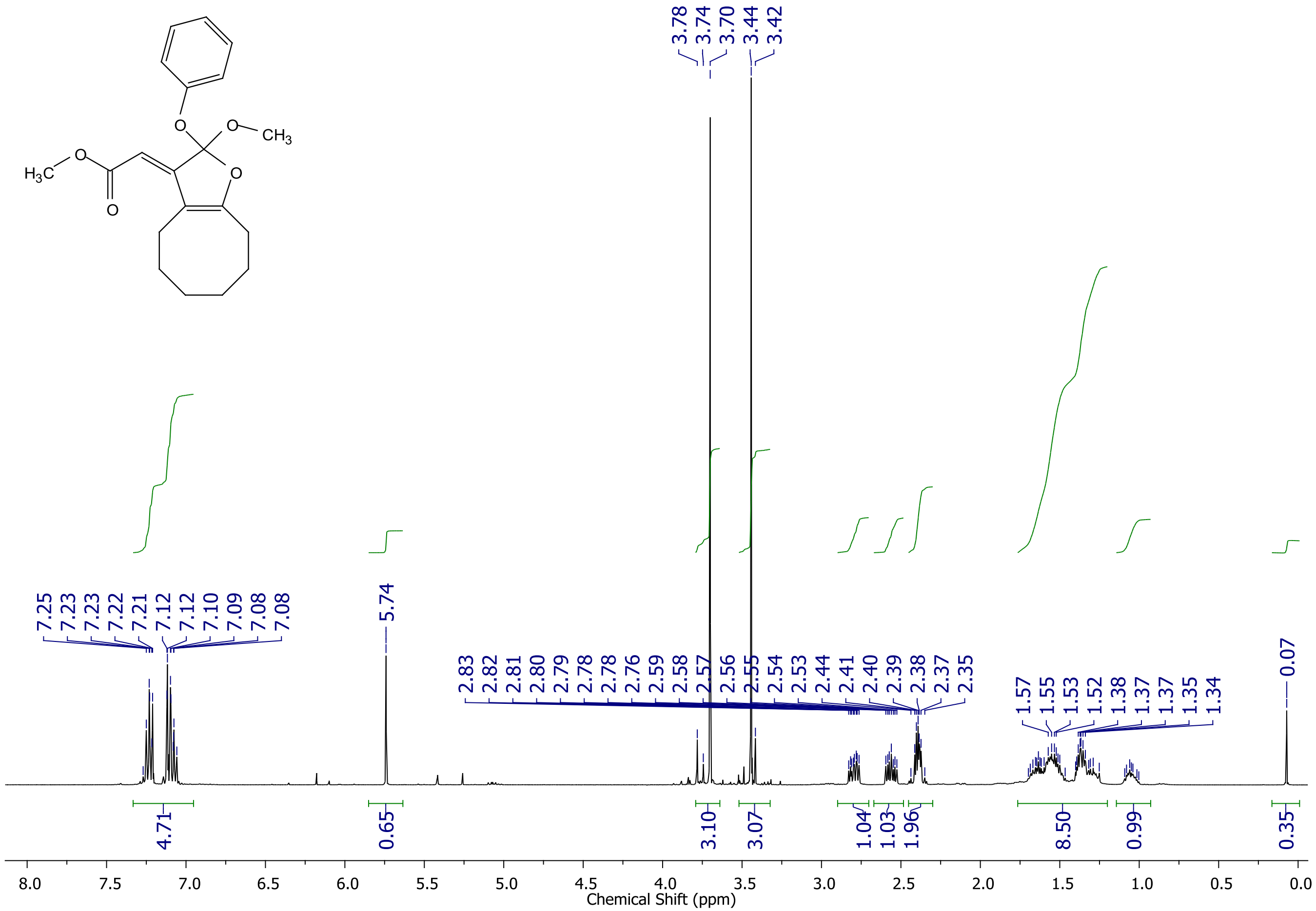

Figure S13. ^{13}C NMR spectrum of **4c**.

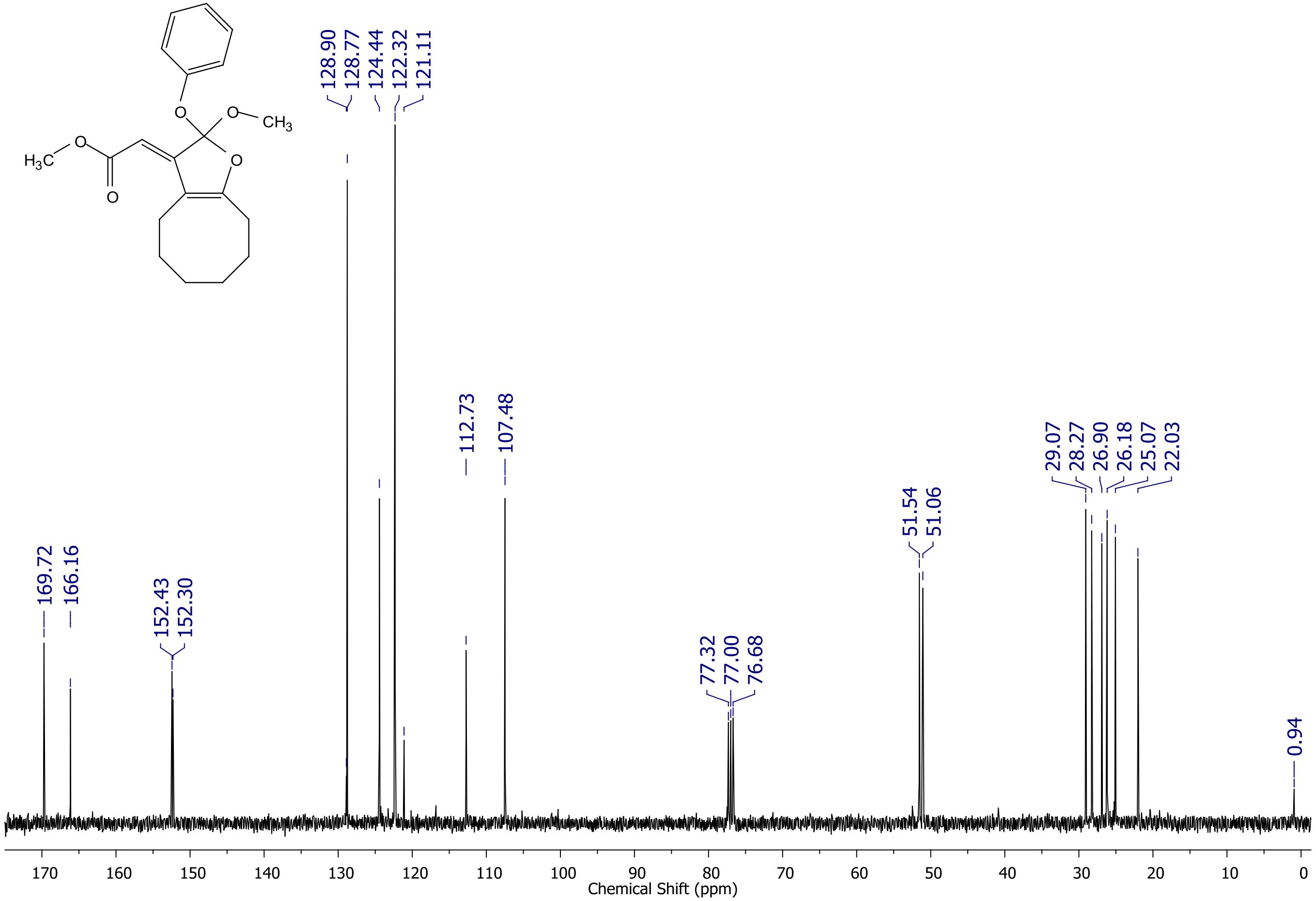

Figure S14. IR spectrum of **4c**.

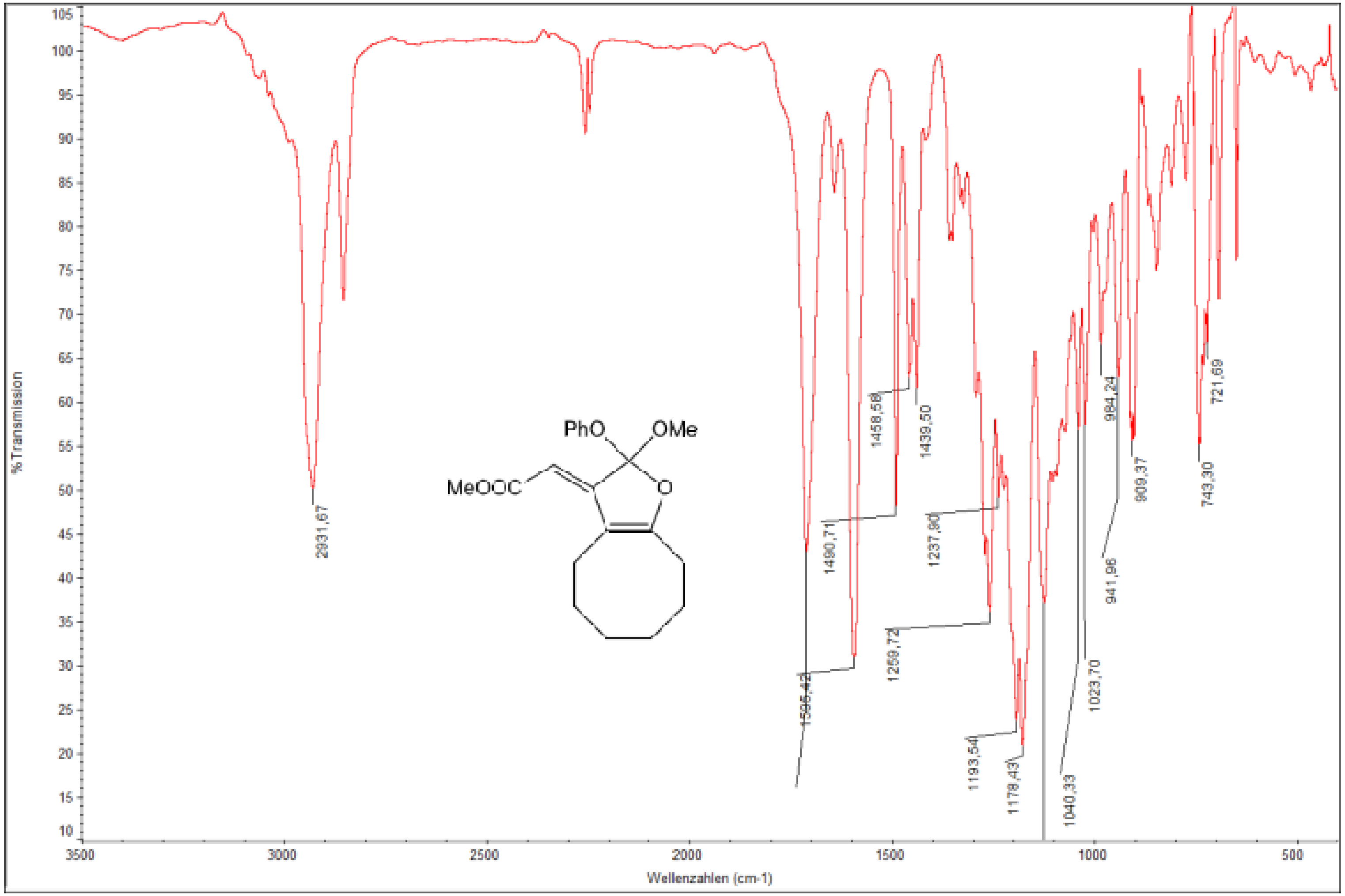

Figure S15. ^{1}H NMR spectrum of (E)-5.

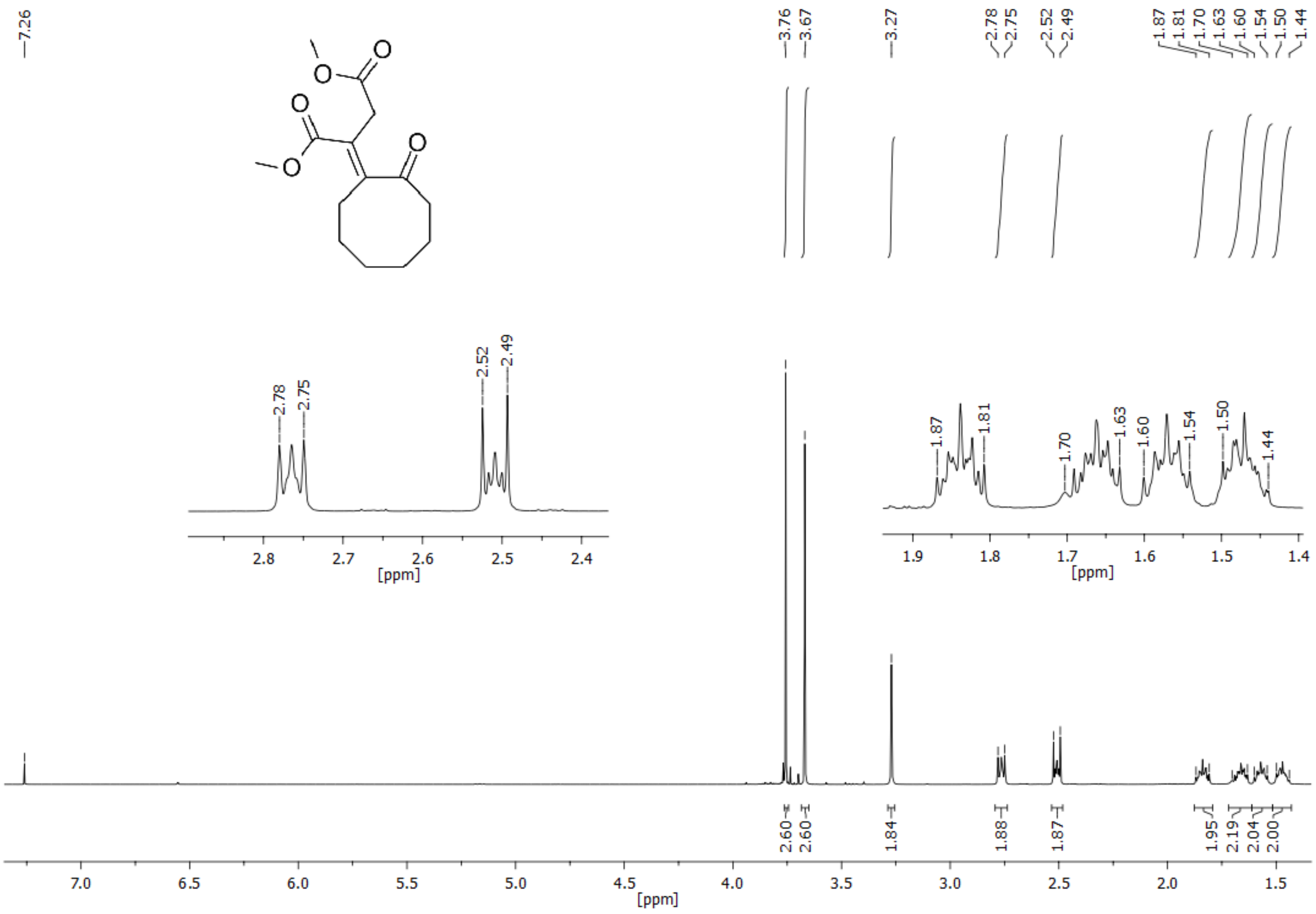

Figure S16. ^{13}C NMR spectrum of (E)-5.

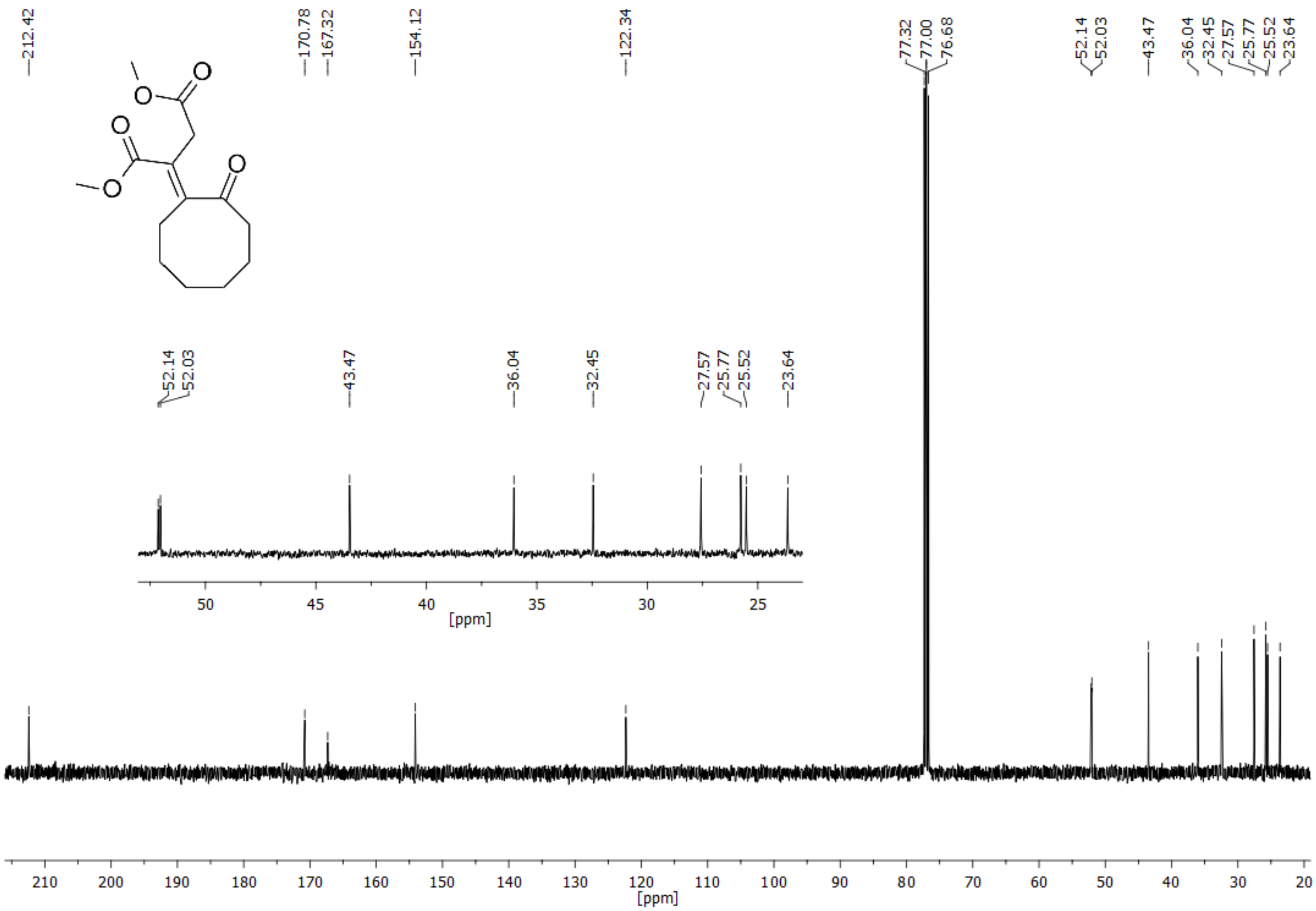

Figure S17. gHSQC-AD spectrum of (E)-5.

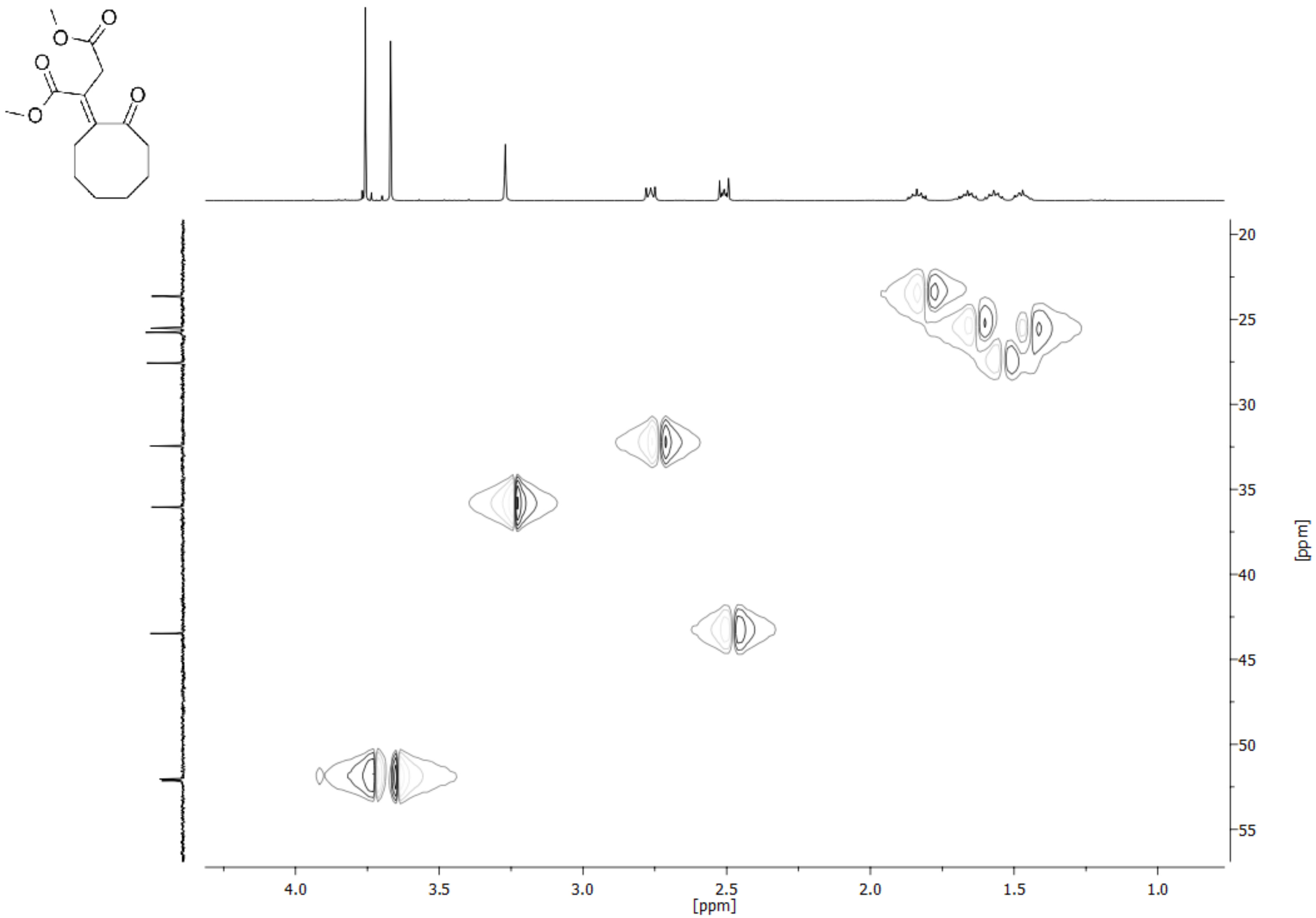

Figure S18. gHMBC-AD spectrum of (E)-5.

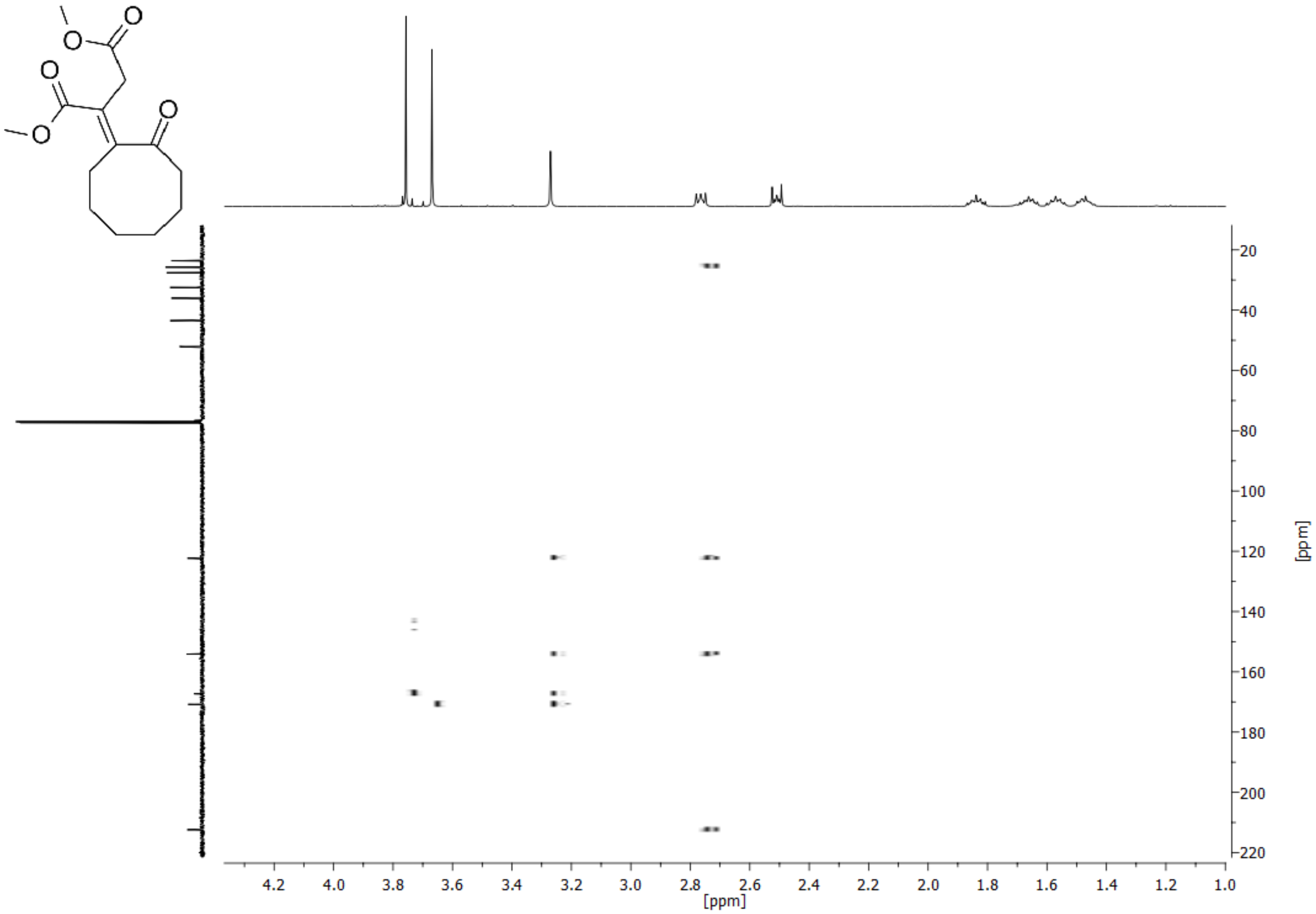

Figure S19. ^{1}H NMR spectrum of (Z)-5.

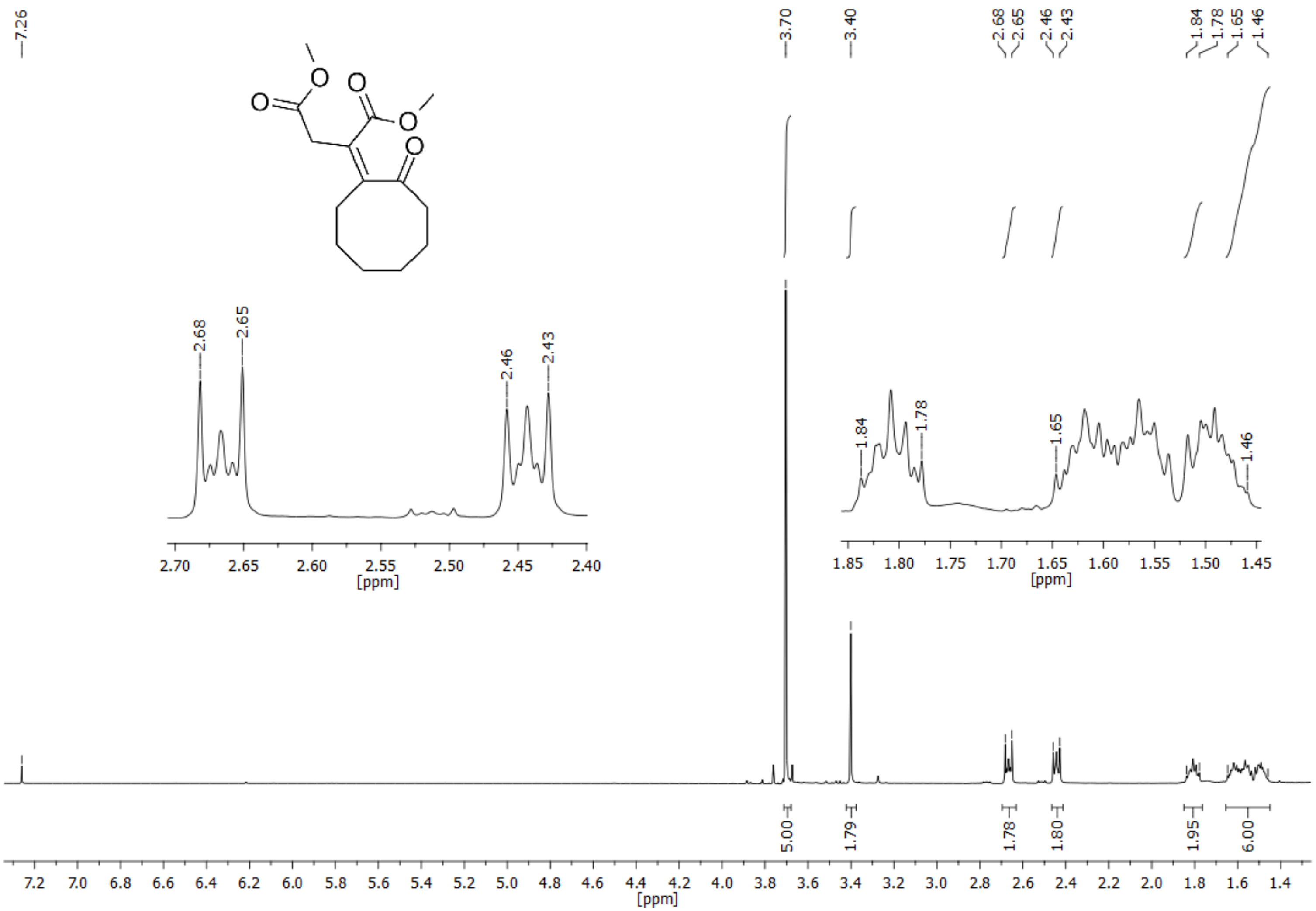

Figure S20. ^{13}C NMR spectrum of (Z)-5.

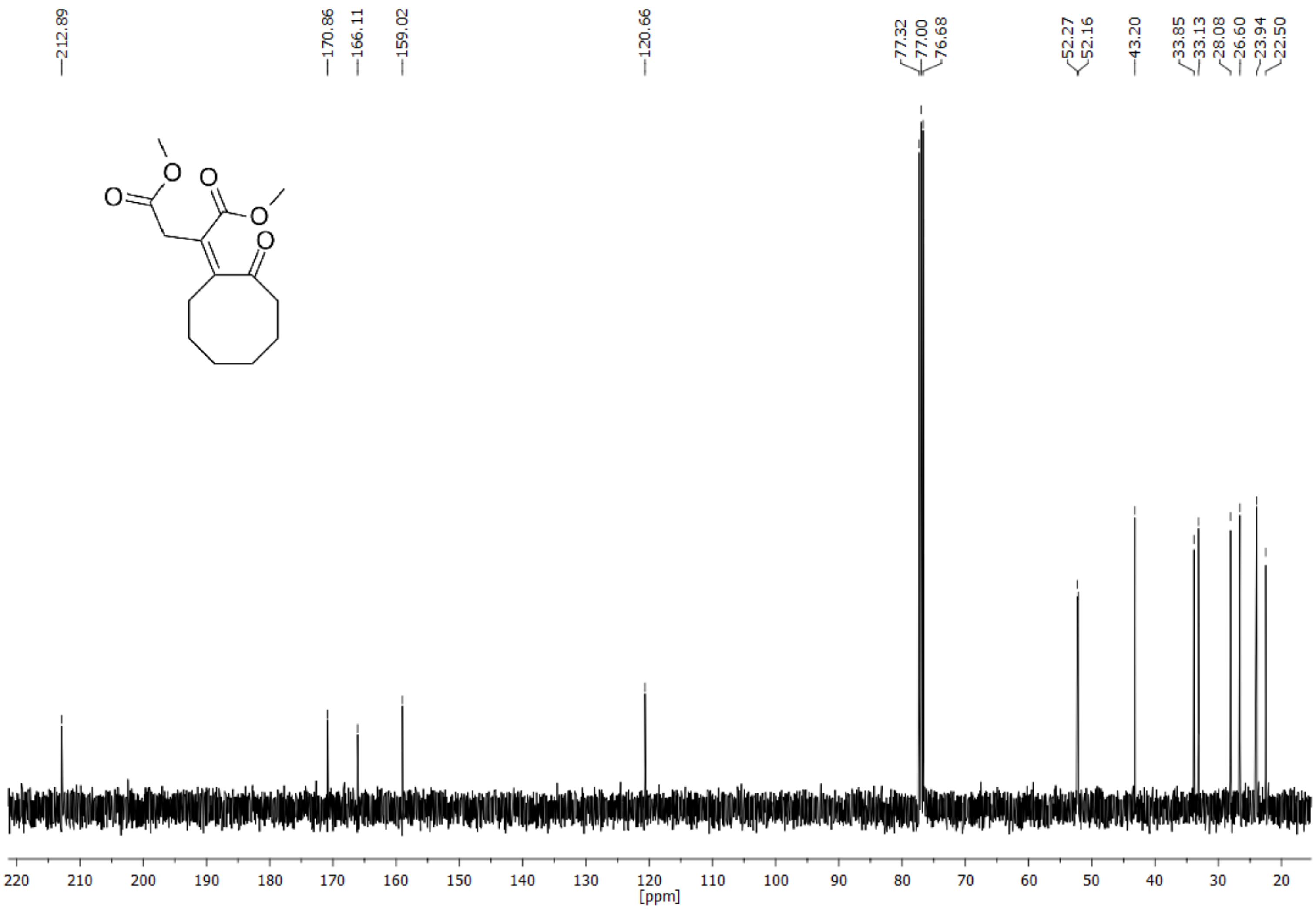

Figure S21. ^{1}H NMR spectrum of **6a**.

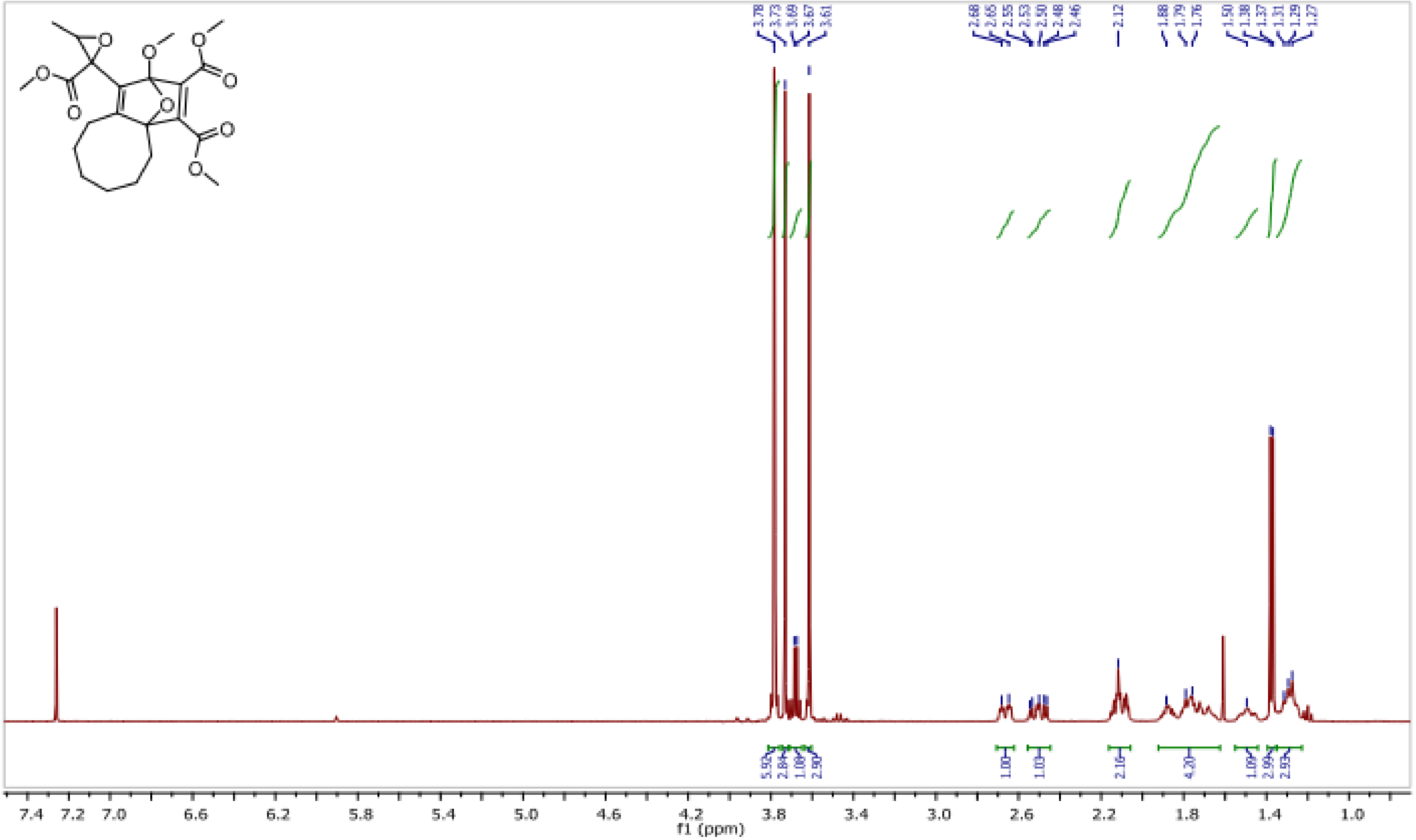

Figure S22. ^{13}C NMR spectrum of **6a**.

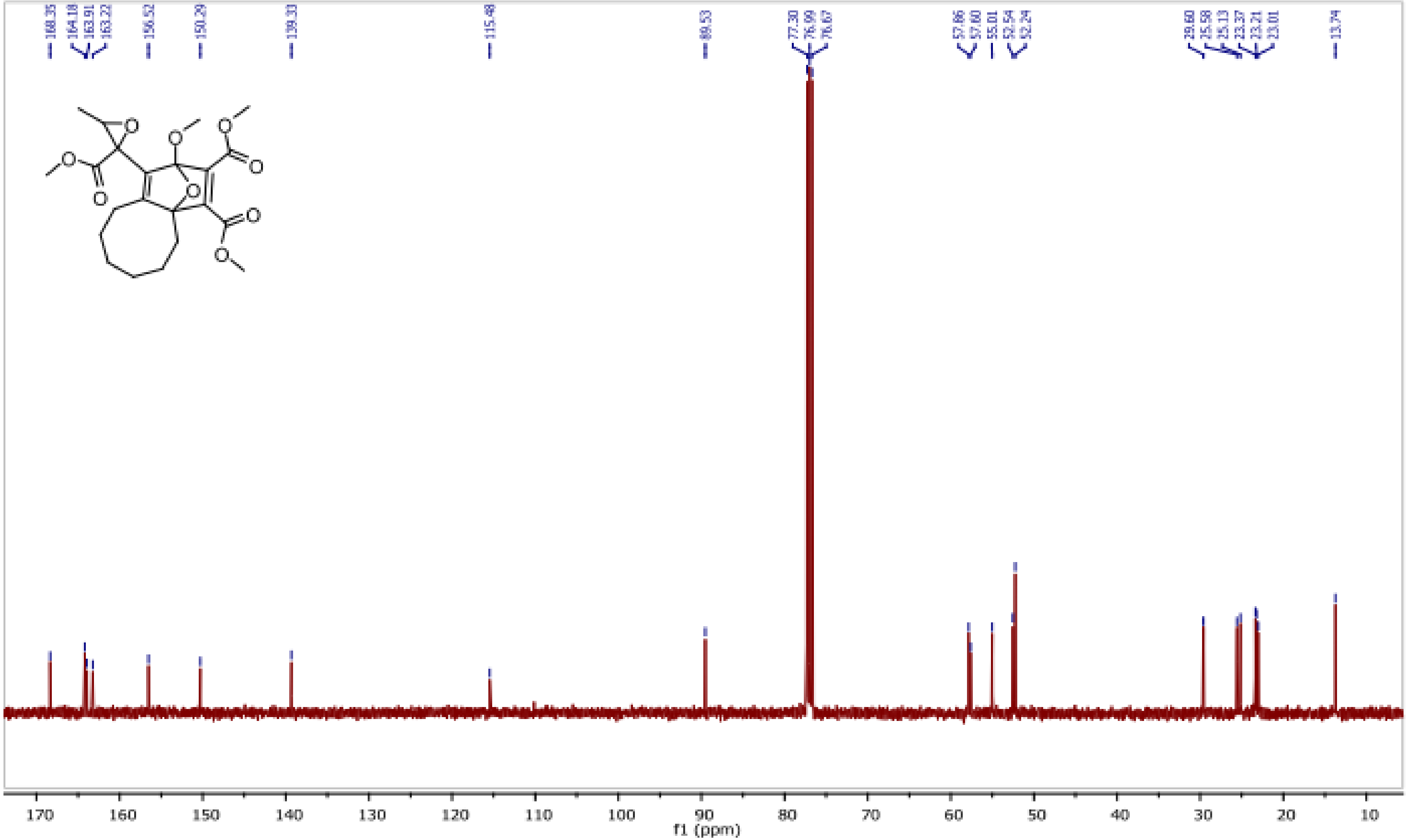

Figure S23. IR spectrum of **6a**.

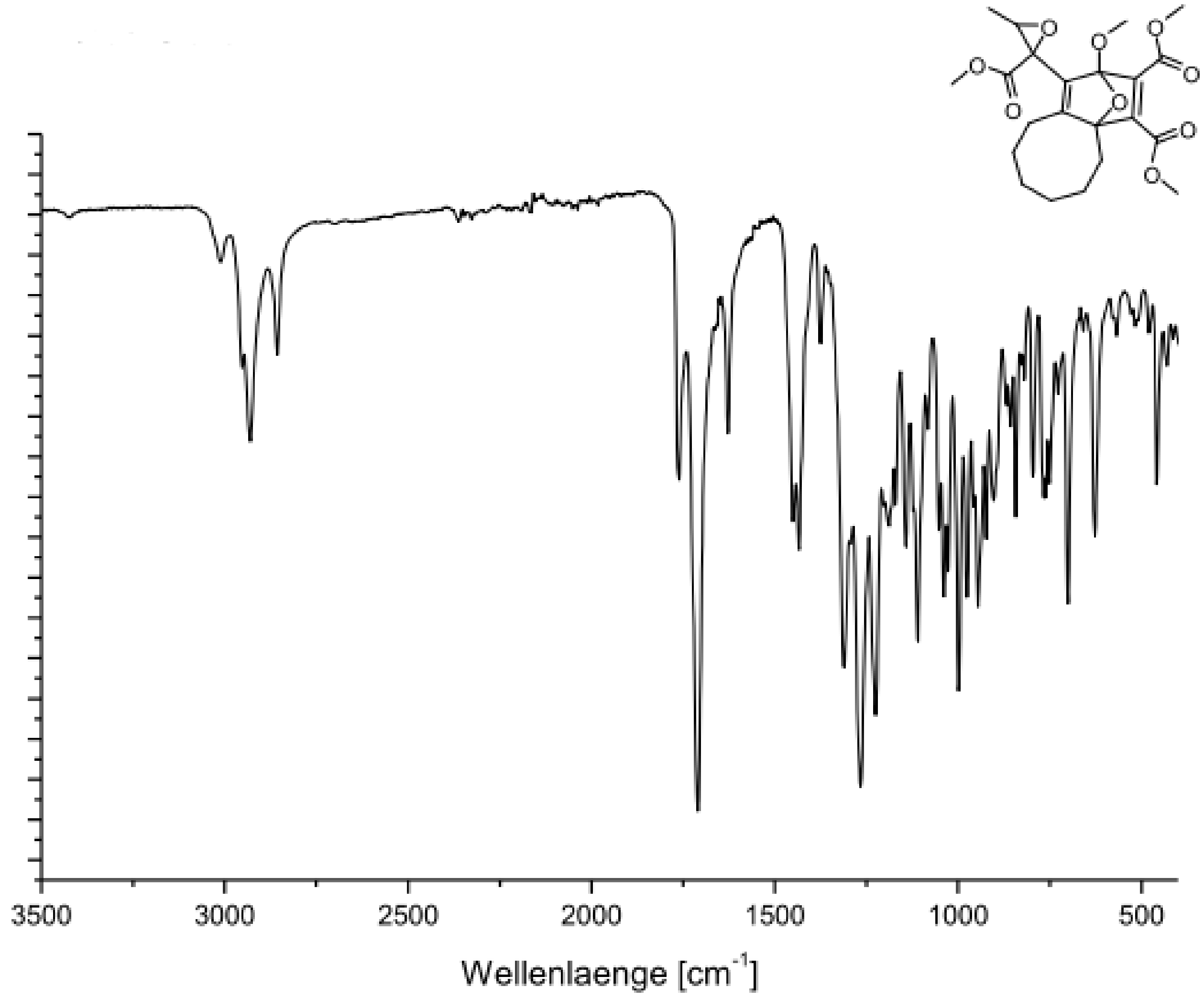

Figure S24. ^{1}H NMR spectrum of **6b**.

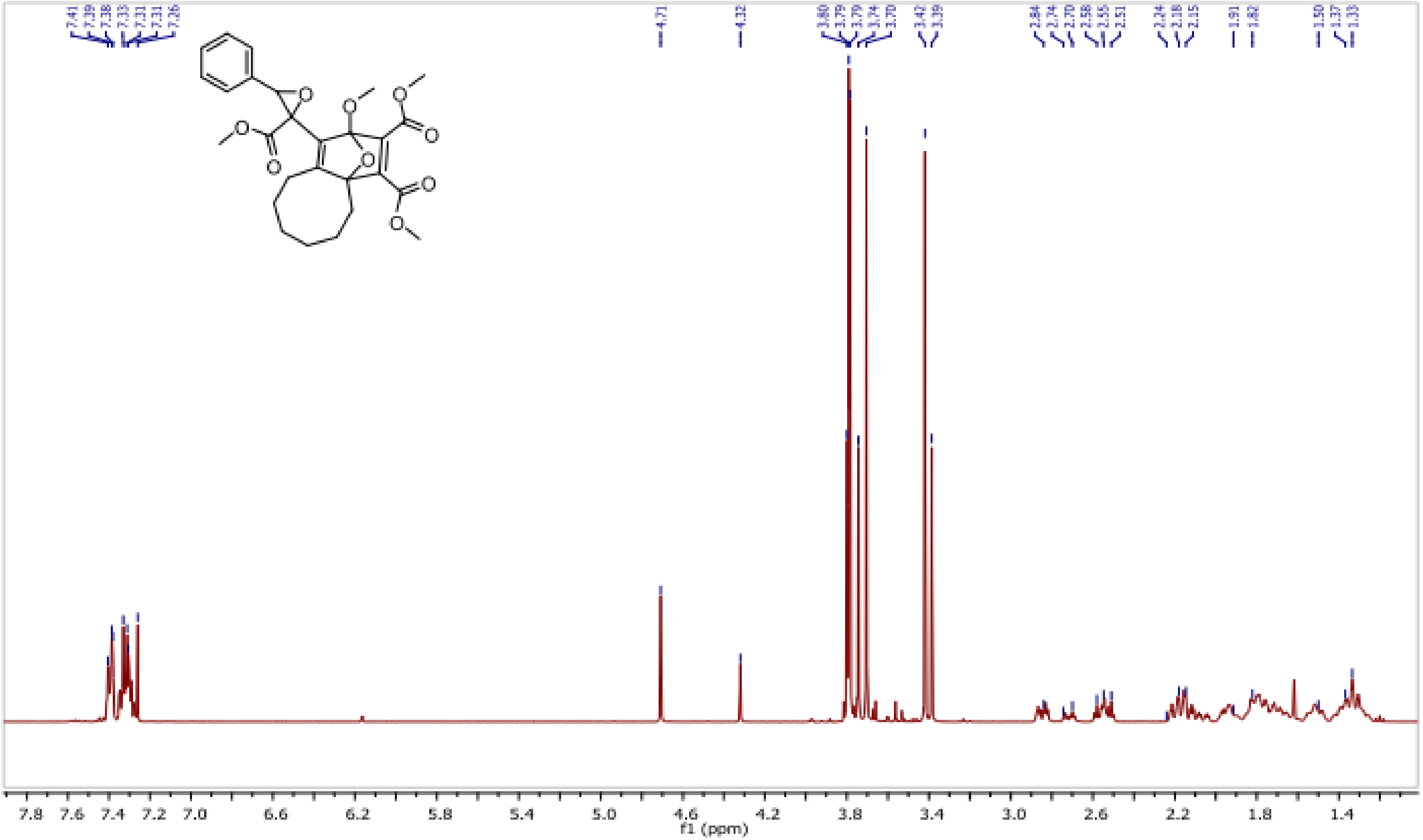

Figure S25. ^{13}C NMR spectrum of **6b**.

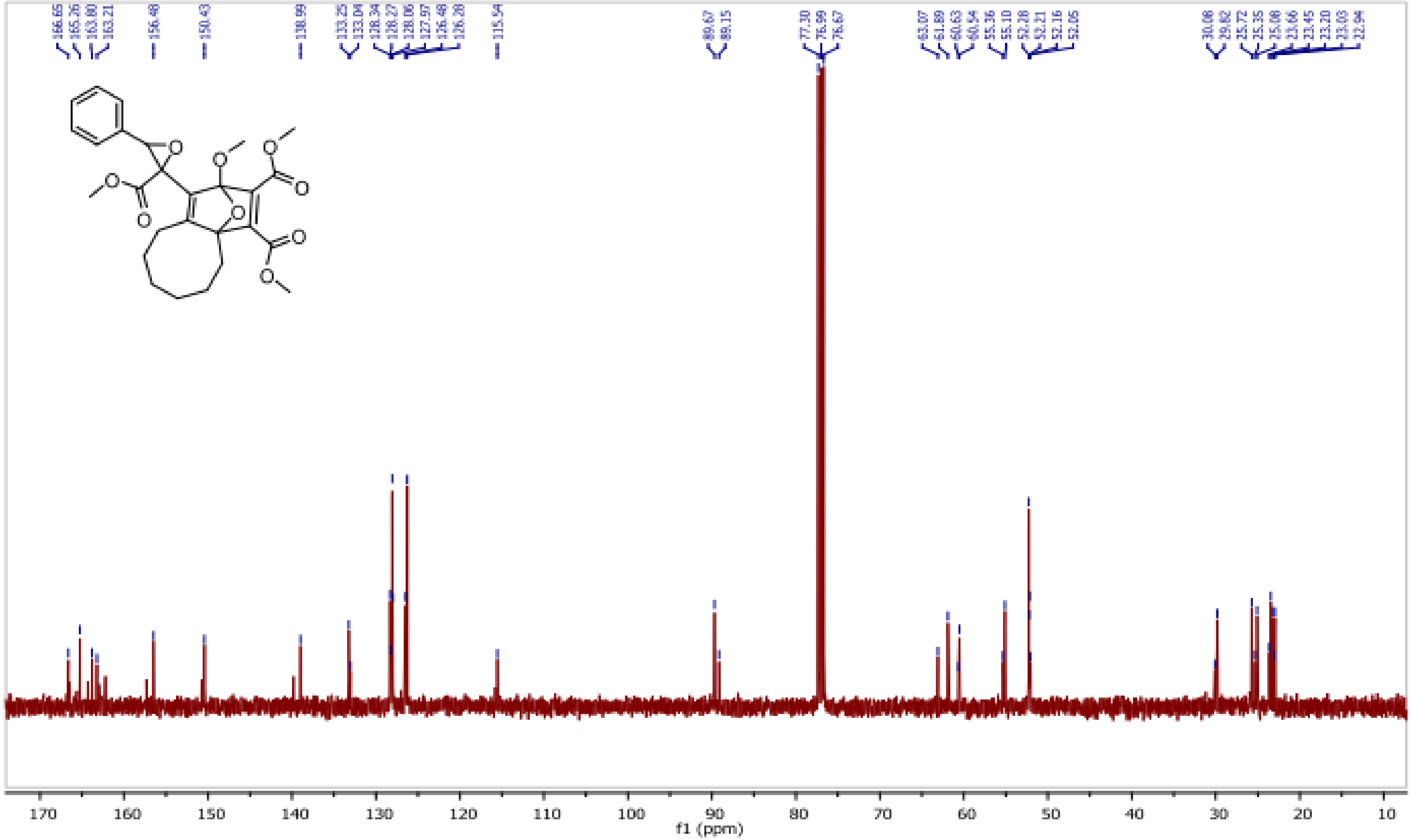

Figure S26. IR spectrum of **6b**.

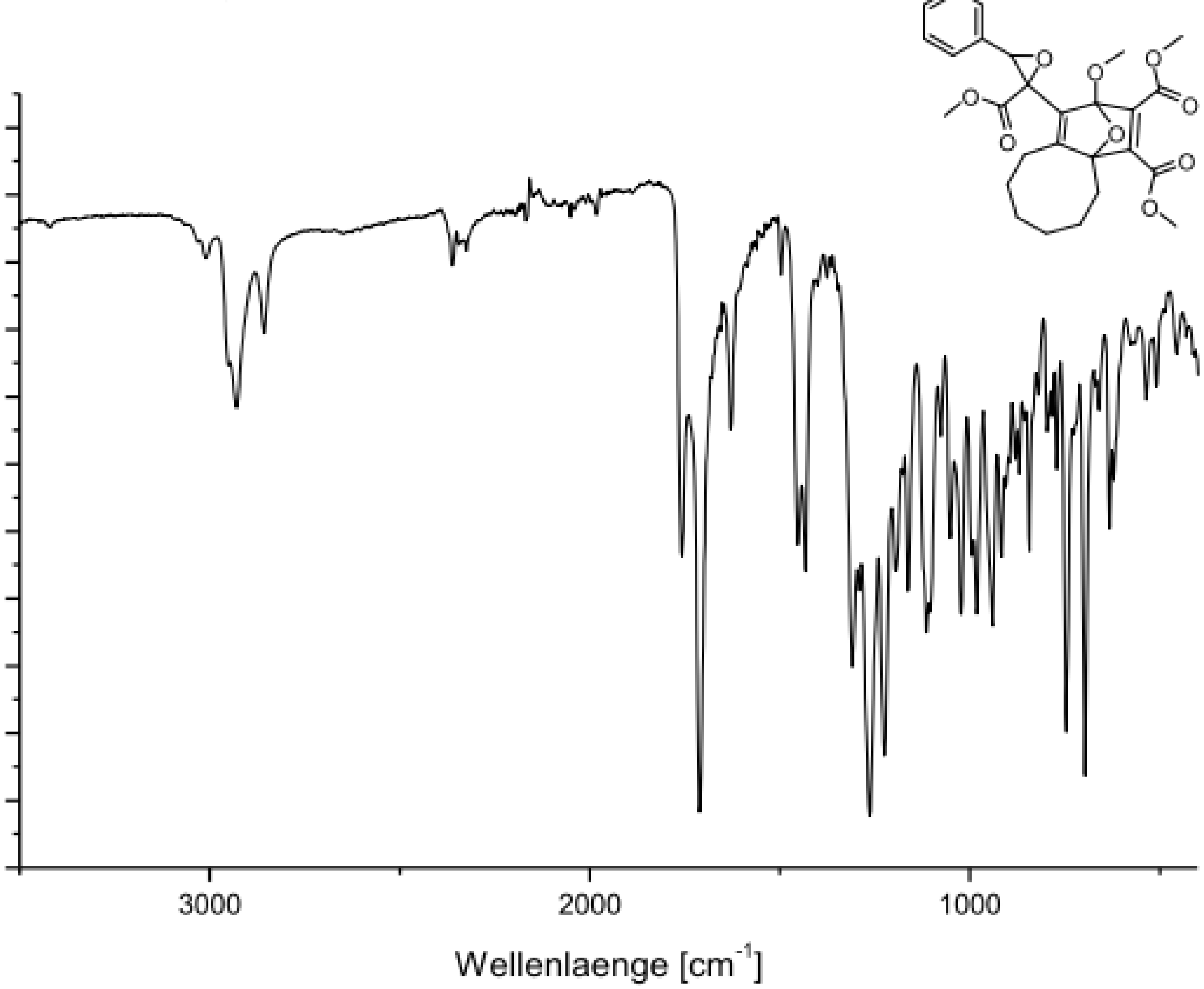

Figure S27. ^{1}H-NMR spectrum of **7**.

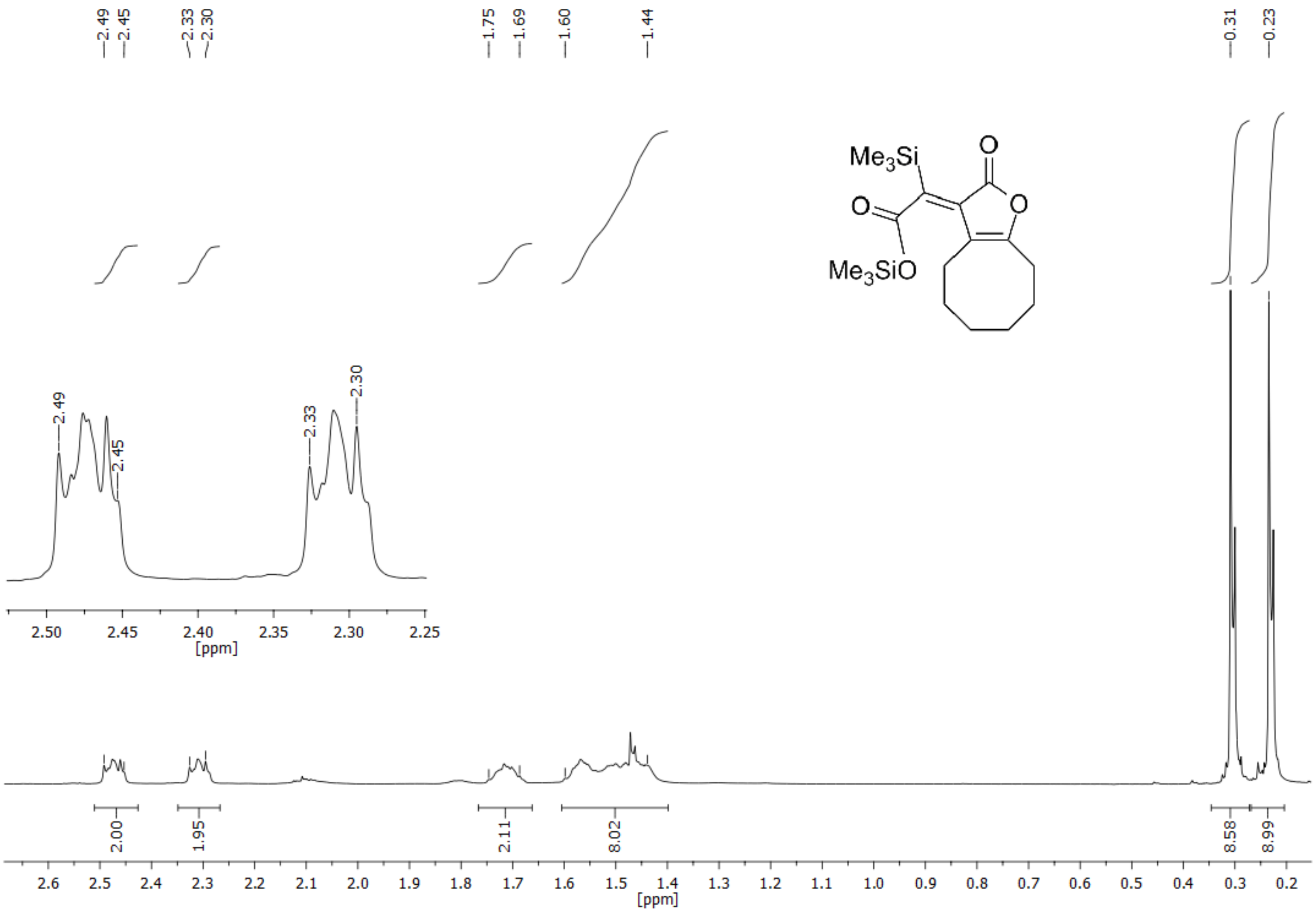

Figure S28. ^{13}C NMR spectrum of **7**.

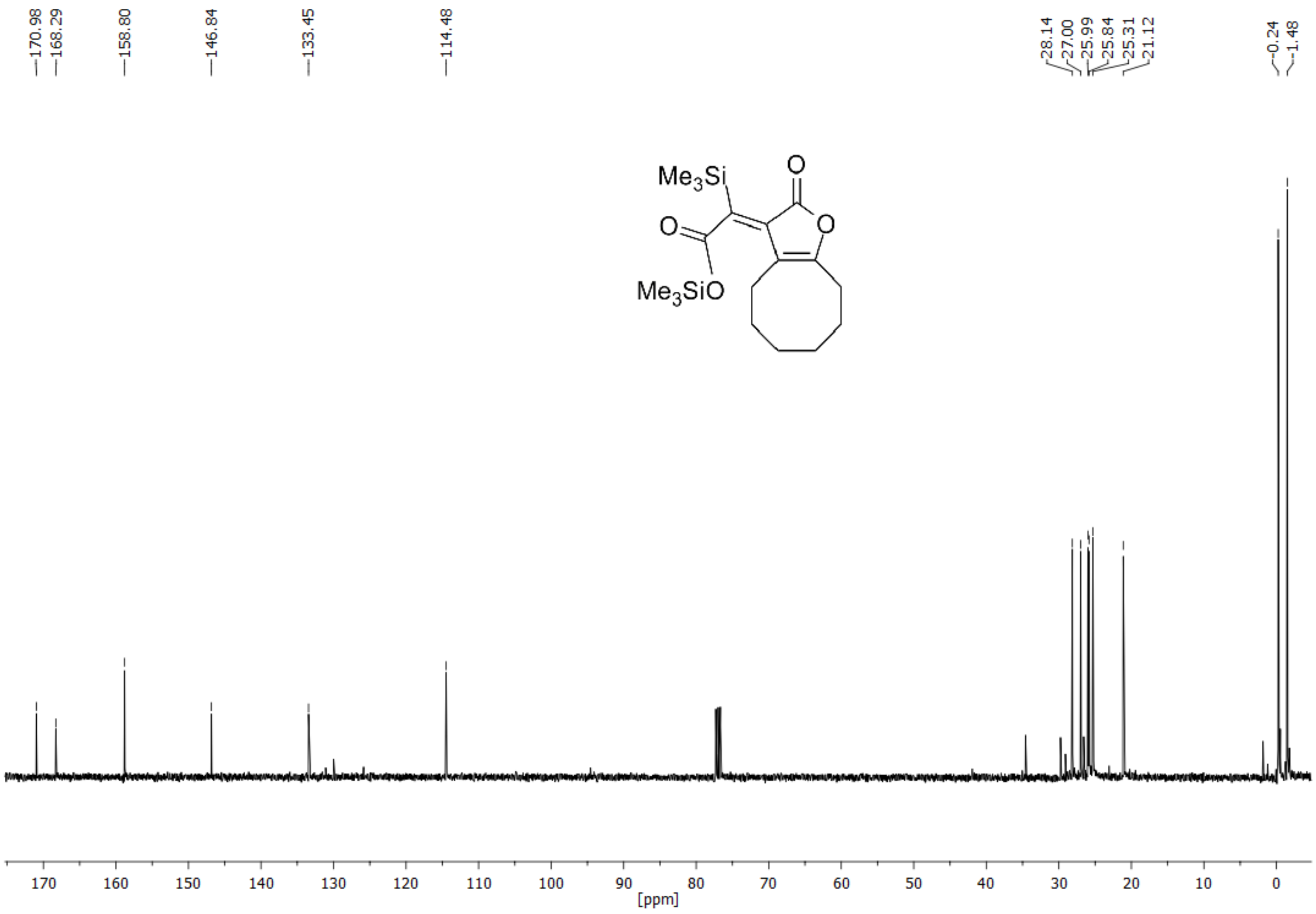

Figure S29. gHMBC-AC spectrum of **7**.

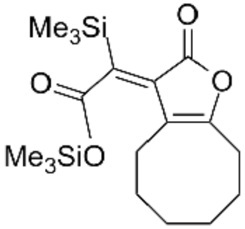

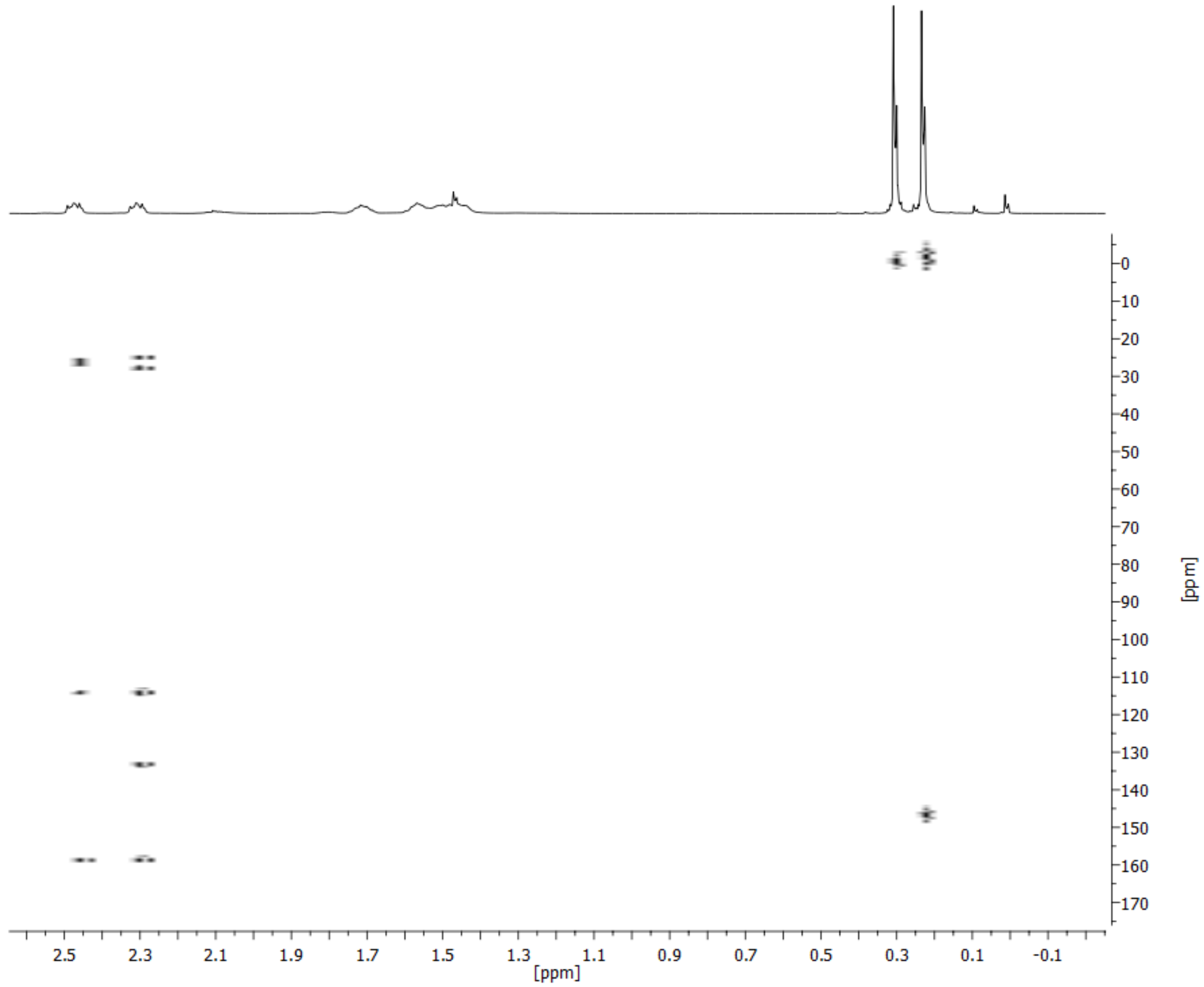

Figure S30. IR spectrum of **7**.

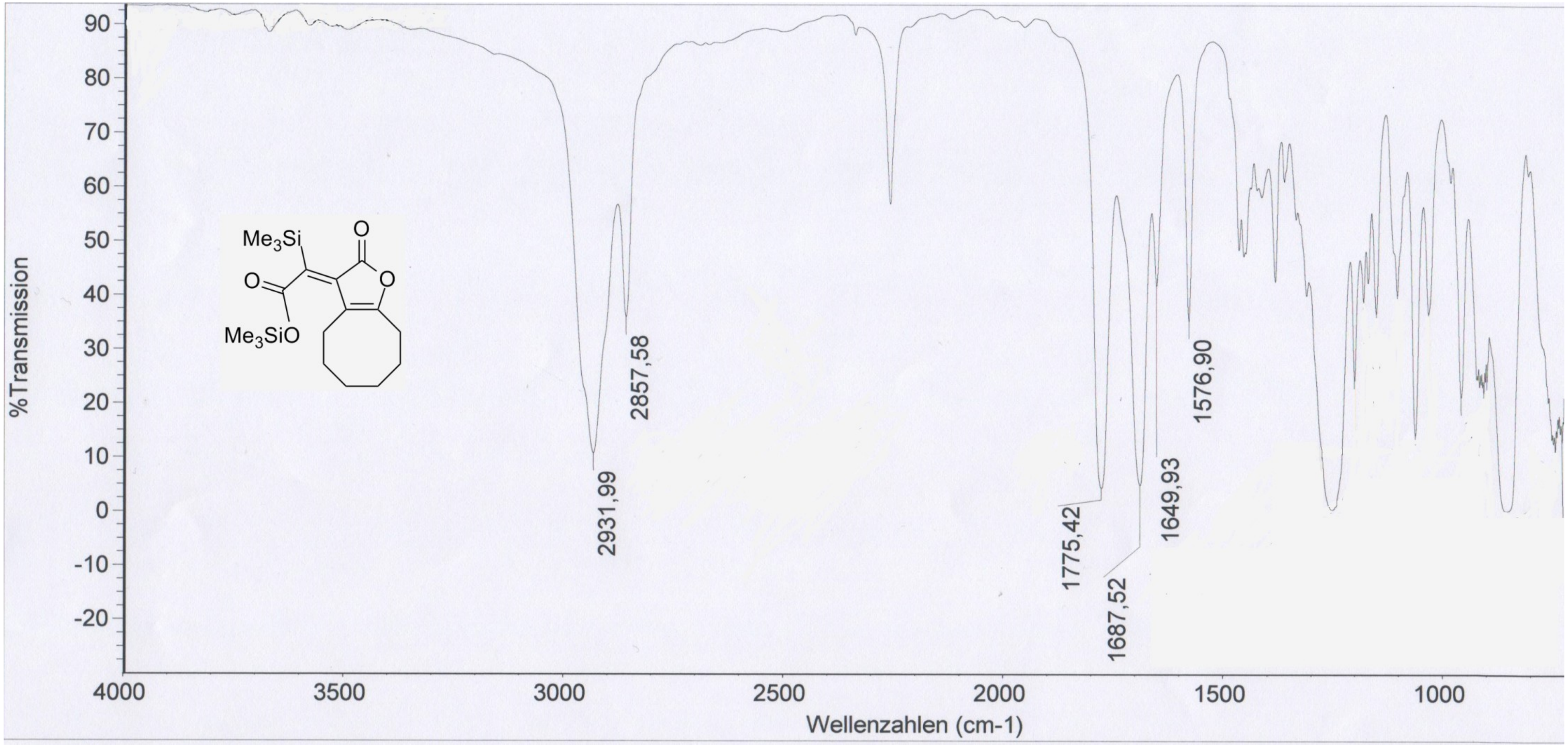

checkCIF/PLATON report

Structure factors have been supplied for datablock(s) ihle01

THIS REPORT IS FOR GUIDANCE ONLY. IF USED AS PART OF A REVIEW PROCEDURE FOR PUBLICATION, IT SHOULD NOT REPLACE THE EXPERTISE OF AN EXPERIENCED CRYSTALLOGRAPHIC REFEREE.

No syntax errors found. CIF dictionary Interpreting this report

Datablock: ihle01

```
Bond precision:  C-C = 0.0020 A          Wavelength=0.71073

Cell:            a=31.6876(13)    b=10.1200(3)       c=16.1580(6)
                 alpha=90         beta=101.909(4)    gamma=90
Temperature:     100 K

                 Calculated                 Reported
Volume           5070.0(3)                  5070.0(3)
Space group      C 2/c                      C2/c
Hall group       -C 2yc                     -C 2yc
Moiety formula   C26 H30 O12                C26 H30 O12
Sum formula      C26 H30 O12                C26 H30 O12
Mr               534.50                     534.50
Dx,g cm-3        1.401                      1.400
Z                8                          8
Mu (mm-1)        0.112                      0.112
F000             2256.0                     2256.0
F000'            2257.47
h,k,lmax         37,12,19                   37,12,19
Nref             4468                       4448
Tmin,Tmax        0.951,0.956                0.937,1.000
Tmin'            0.951

Correction method= MULTI-SCAN

Data completeness= 0.996          Theta(max)= 25.000

R(reflections)= 0.0370( 3756)     wR2(reflections)= 0.0909( 4448)

S = 1.032                 Npar= 343
```

The following ALERTS were generated. Each ALERT has the format
test-name_ALERT_alert-type_alert-level.
Click on the hyperlinks for more details of the test.

Alert level G

```
PLAT005_ALERT_5_G No _iucr_refine_instructions_details  in the CIF     Please Do !
PLAT793_ALERT_4_G The Model has Chirality at C8      .............          S Verify
```

```
   0 ALERT level A = Most likely a serious problem - resolve or explain
   0 ALERT level B = A potentially serious problem, consider carefully
   0 ALERT level C = Check. Ensure it is not caused by an omission or oversight
   2 ALERT level G = General information/check it is not something unexpected

   0 ALERT type 1 CIF construction/syntax error, inconsistent or missing data
   0 ALERT type 2 Indicator that the structure model may be wrong or deficient
   0 ALERT type 3 Indicator that the structure quality may be low
   1 ALERT type 4 Improvement, methodology, query or suggestion
   1 ALERT type 5 Informative message, check
```

It is advisable to attempt to resolve as many as possible of the alerts in all categories. Often the minor alerts point to easily fixed oversights, errors and omissions in your CIF or refinement strategy, so attention to these fine details can be worthwhile. In order to resolve some of the more serious problems it may be necessary to carry out additional measurements or structure refinements. However, the purpose of your study may justify the reported deviations and the more serious of these should normally be commented upon in the discussion or experimental section of a paper or in the "special_details" fields of the CIF. checkCIF was carefully designed to identify outliers and unusual parameters, but every test has its limitations and alerts that are not important in a particular case may appear. Conversely, the absence of alerts does not guarantee there are no aspects of the results needing attention. It is up to the individual to critically assess their own results and, if necessary, seek expert advice.

Publication of your CIF in IUCr journals

A basic structural check has been run on your CIF. These basic checks will be run on all CIFs submitted for publication in IUCr journals (*Acta Crystallographica*, *Journal of Applied Crystallography*, *Journal of Synchrotron Radiation*); however, if you intend to submit to *Acta Crystallographica Section C* or *E*, you should make sure that full publication checks are run on the final version of your CIF prior to submission.

Publication of your CIF in other journals

Please refer to the *Notes for Authors* of the relevant journal for any special instructions relating to CIF submission.

PLATON version of 24/07/2014; check.def file version of 24/07/2014

Datablock ihle01 - ellipsoid plot

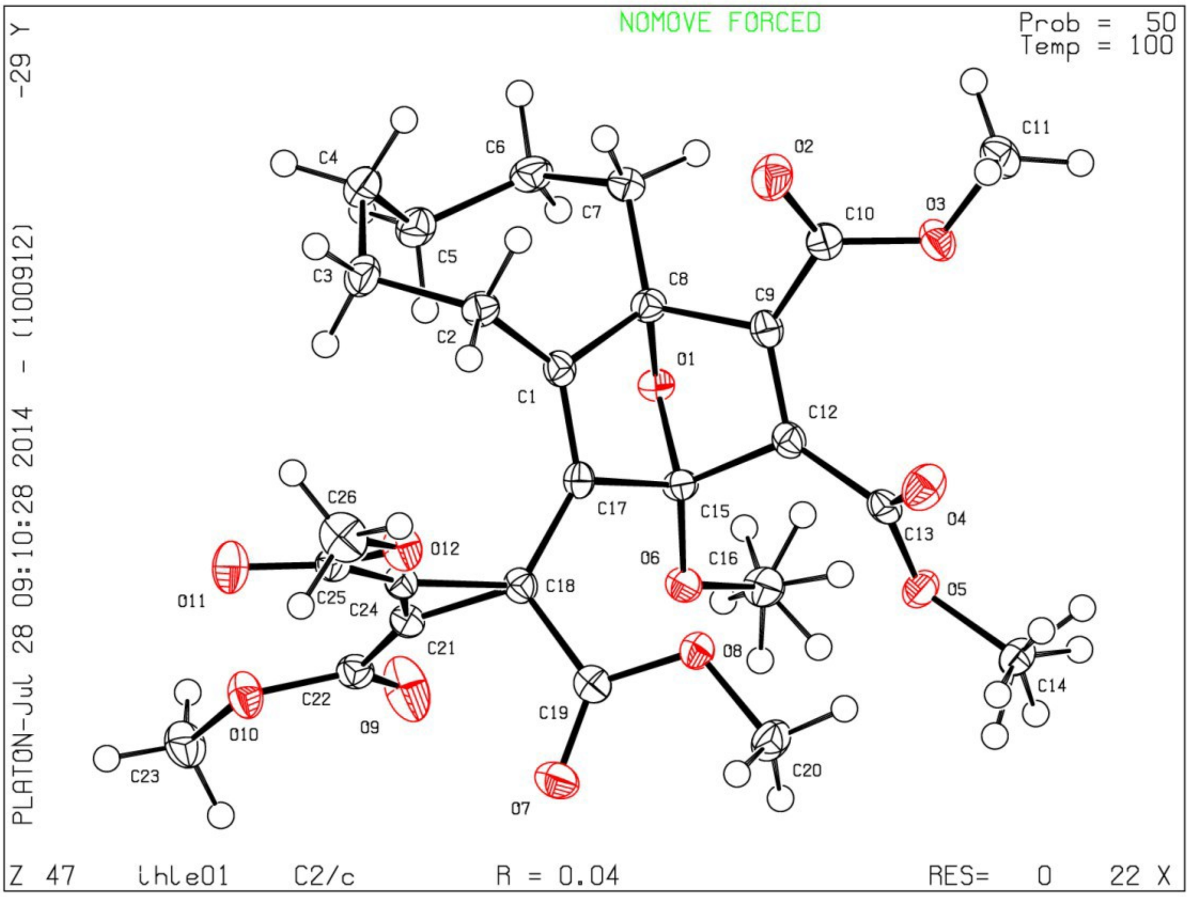

3b

checkCIF/PLATON report

Structure factors have been supplied for datablock(s) shelx

THIS REPORT IS FOR GUIDANCE ONLY. IF USED AS PART OF A REVIEW PROCEDURE FOR PUBLICATION, IT SHOULD NOT REPLACE THE EXPERTISE OF AN EXPERIENCED CRYSTALLOGRAPHIC REFEREE.

No syntax errors found. CIF dictionary Interpreting this report

Datablock: shelx

```
Bond precision:   C-C = 0.0027 A          Wavelength=1.54184

Cell:            a=18.1382(3)     b=11.3690(2)        c=15.9193(4)
                 alpha=90         beta=118.261(2)     gamma=90
Temperature:     110 K

                 Calculated                   Reported
Volume           2891.46(11)                  2891.44(12)
Space group      I 2/a                        I 2/a
Hall group       -I 2ya                       -I 2ya
Moiety formula   C15 H22 O5                   C15 H22 O5
Sum formula      C15 H22 O5                   C15 H22 O5
Mr               282.33                       282.32
Dx,g cm-3        1.297                        1.297
Z                8                            8
Mu (mm-1)        0.798                        0.798
F000             1216.0                       1216.0
F000'            1220.01
h,k,lmax         20,12,18                     20,13,18
Nref             2263                         2243
Tmin,Tmax        0.852,0.887                  0.933,1.000
Tmin'            0.852

Correction method= MULTI-SCAN

Data completeness= 0.991          Theta(max)= 61.986

R(reflections)= 0.0430( 2096)     wR2(reflections)= 0.1190( 2243)

S = 1.033                   Npar= 181
```

The following ALERTS were generated. Each ALERT has the format
test-name_ALERT_alert-type_alert-level.
Click on the hyperlinks for more details of the test.

Alert level B

```
                Crystal system given = monoclinic
THETM01_ALERT_3_B  The value of sine(theta_max)/wavelength is less than 0.575
                Calculated sin(theta_max)/wavelength =    0.5726
```

Alert level G

```
PLAT128_ALERT_4_G Alternate Setting for Input Space Group  I2/a       C2/c    Note
```

```
   0 ALERT level A = Most likely a serious problem - resolve or explain
   1 ALERT level B = A potentially serious problem, consider carefully
   0 ALERT level C = Check. Ensure it is not caused by an omission or oversight
   1 ALERT level G = General information/check it is not something unexpected

   0 ALERT type 1 CIF construction/syntax error, inconsistent or missing data
   0 ALERT type 2 Indicator that the structure model may be wrong or deficient
   1 ALERT type 3 Indicator that the structure quality may be low
   1 ALERT type 4 Improvement, methodology, query or suggestion
   0 ALERT type 5 Informative message, check
```

It is advisable to attempt to resolve as many as possible of the alerts in all categories. Often the minor alerts point to easily fixed oversights, errors and omissions in your CIF or refinement strategy, so attention to these fine details can be worthwhile. In order to resolve some of the more serious problems it may be necessary to carry out additional measurements or structure refinements. However, the purpose of your study may justify the reported deviations and the more serious of these should normally be commented upon in the discussion or experimental section of a paper or in the "special_details" fields of the CIF. checkCIF was carefully designed to identify outliers and unusual parameters, but every test has its limitations and alerts that are not important in a particular case may appear. Conversely, the absence of alerts does not guarantee there are no aspects of the results needing attention. It is up to the individual to critically assess their own results and, if necessary, seek expert advice.

Publication of your CIF in IUCr journals

A basic structural check has been run on your CIF. These basic checks will be run on all CIFs submitted for publication in IUCr journals (*Acta Crystallographica*, *Journal of Applied Crystallography*, *Journal of Synchrotron Radiation*); however, if you intend to submit to *Acta Crystallographica Section C* or *E*, you should make sure that full publication checks are run on the final version of your CIF prior to submission.

Publication of your CIF in other journals

Please refer to the *Notes for Authors* of the relevant journal for any special instructions relating to CIF submission.

PLATON version of 24/07/2014; check.def file version of 24/07/2014

Datablock shelx - ellipsoid plot

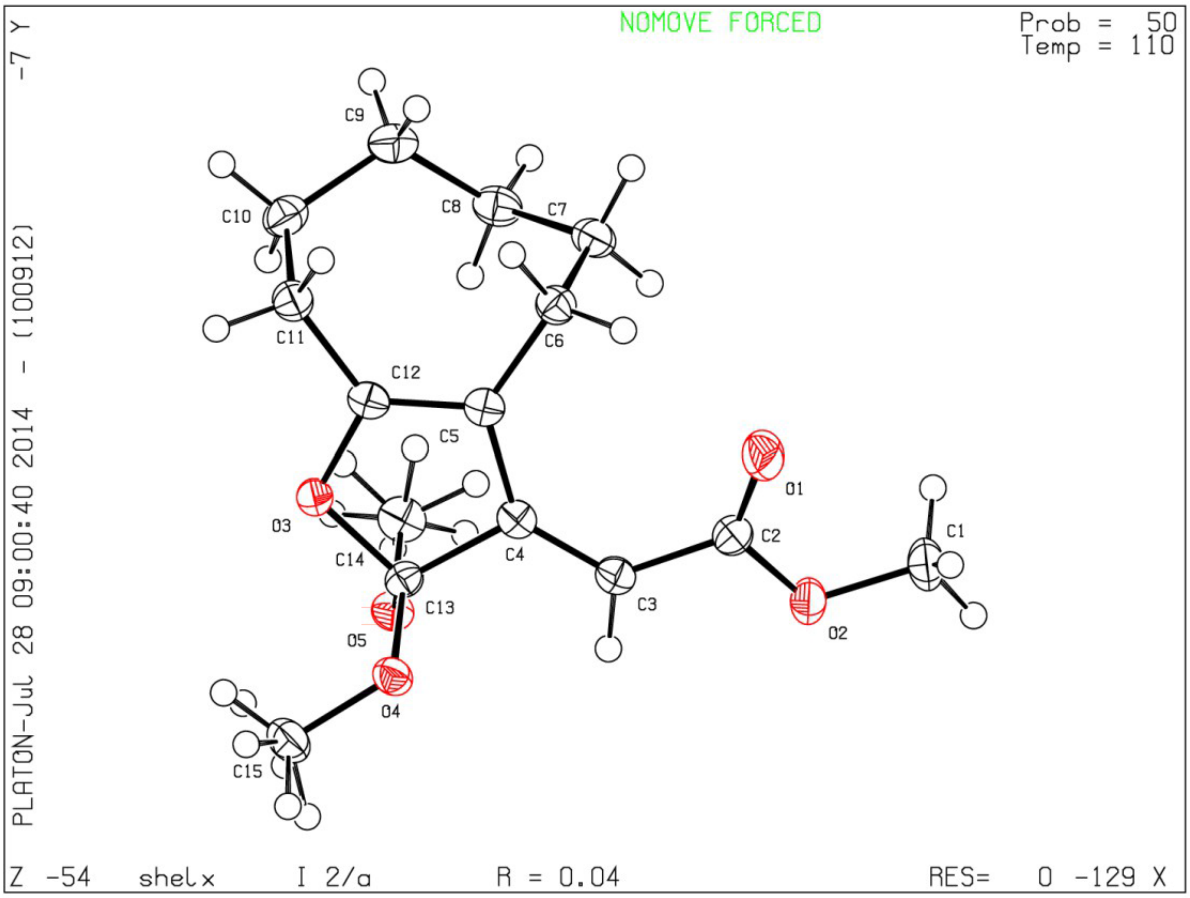

4a

checkCIF/PLATON (standard)

Structure factors have been supplied for datablock(s) shelxl

THIS REPORT IS FOR GUIDANCE ONLY. IF USED AS PART OF A REVIEW PROCEDURE FOR PUBLICATION, IT SHOULD NOT REPLACE THE EXPERTISE OF AN EXPERIENCED CRYSTALLOGRAPHIC REFEREE.

No syntax errors found. CIF dictionary
Please wait while processing .... Interpreting this report

Datablock: shelxl

Bond precision: C-C = 0.0041 A Wavelength=1.54184

Cell: a=8.8577(4) b=28.5456(16) c=17.3589(9)
alpha=90 beta=90.063(4) gamma=90

Temperature: 100 K

	Calculated	Reported
Volume	4389.2(4)	4389.2(4)
Space group	P 21/c	P 21/c
Hall group	-P 2ybc	?
Moiety formula	C22 H28 O9	C22 H28 O9
Sum formula	C22 H28 O9	C22 H28 O9
Mr	436.44	436.44
Dx,g cm-3	1.321	1.321
Z	8	8
Mu (mm-1)	0.863	0.863
F000	1856.0	1856.0
F000'	1862.54	
h,k,lmax	10,32,19	10,32,19
Nref	6852	6811
Tmin,Tmax	0.902,0.991	0.724,0.991
Tmin'	0.708	

Correction method= MULTI-SCAN

Data completeness= 0.994 Theta(max)= 61.770

R(reflections)= 0.0548(5141) wR2(reflections)= 0.1618(6811)

S = 1.068 Npar= 559

The following ALERTS were generated. Each ALERT has the format
test-name_ALERT_alert-type_alert-level.
Click on the hyperlinks for more details of the test.

Alert level B

THETM01_ALERT_3_B The value of sine(theta_max)/wavelength is less than 0.575
Calculated sin(theta_max)/wavelength = 0.5714

Alert level C

PLAT340_ALERT_3_C Low Bond Precision on C-C Bonds .... 0.0041 Ang.
PLAT413_ALERT_2_C Short Inter XH3 .. XHn H35B .. H44A .. 2.11 Ang.

Alert level G

PLAT005_ALERT_5_G No _iucr_refine_instructions_details in the CIF Please Do !
PLAT793_ALERT_4_G The Model has Chirality at C1 .... R Verify
And 6 other PLAT793 Alerts
More ...

0 **ALERT level A** = Most likely a serious problem - resolve or explain
1 **ALERT level B** = A potentially serious problem, consider carefully
2 **ALERT level C** = Check. Ensure it is not caused by an omission or oversight
8 **ALERT level G** = General information/check it is not something unexpected

0 ALERT type 1 CIF construction/syntax error, inconsistent or missing data
1 ALERT type 2 Indicator that the structure model may be wrong or deficient
2 ALERT type 3 Indicator that the structure quality may be low
7 ALERT type 4 Improvement, methodology, query or suggestion
1 ALERT type 5 Informative message, check

It is advisable to attempt to resolve as many as possible of the alerts in all categories. Often the minor alerts point to easily fixed oversights, errors and omissions in your CIF or refinement strategy, so attention to these fine details can be worthwhile. In order to resolve some of the more serious problems it may be necessary to carry out

additional measurements or structure refinements. However, the purpose of your study may justify the reported deviations and the more serious of these should normally be commented upon in the discussion or experimental section of a paper or in the "special_details" fields of the CIF. checkCIF was carefully designed to identify outliers and unusual parameters, but every test has its limitations and alerts that are not important in a particular case may appear. Conversely, the absence of alerts does not guarantee there are no aspects of the results needing attention. It is up to the individual to critically assess their own results and, if necessary, seek expert advice.

Publication of your CIF in IUCr journals

A basic structural check has been run on your CIF. These basic checks will be run on all CIFs submitted for publication in IUCr journals (*Acta Crystallographica*, *Journal of Applied Crystallography*, *Journal of Synchrotron Radiation*); however, if you intend to submit to *Acta Crystallographica Section C* or *E*, you should make sure that full publication checks are run on the final version of your CIF prior to submission.

Publication of your CIF in other journals

Please refer to the *Notes for Authors* of the relevant journal for any special instructions relating to CIF submission.

PLATON version of 24/07/2014; check.def file version of 24/07/2014

Datablock shelxl - ellipsoid plot

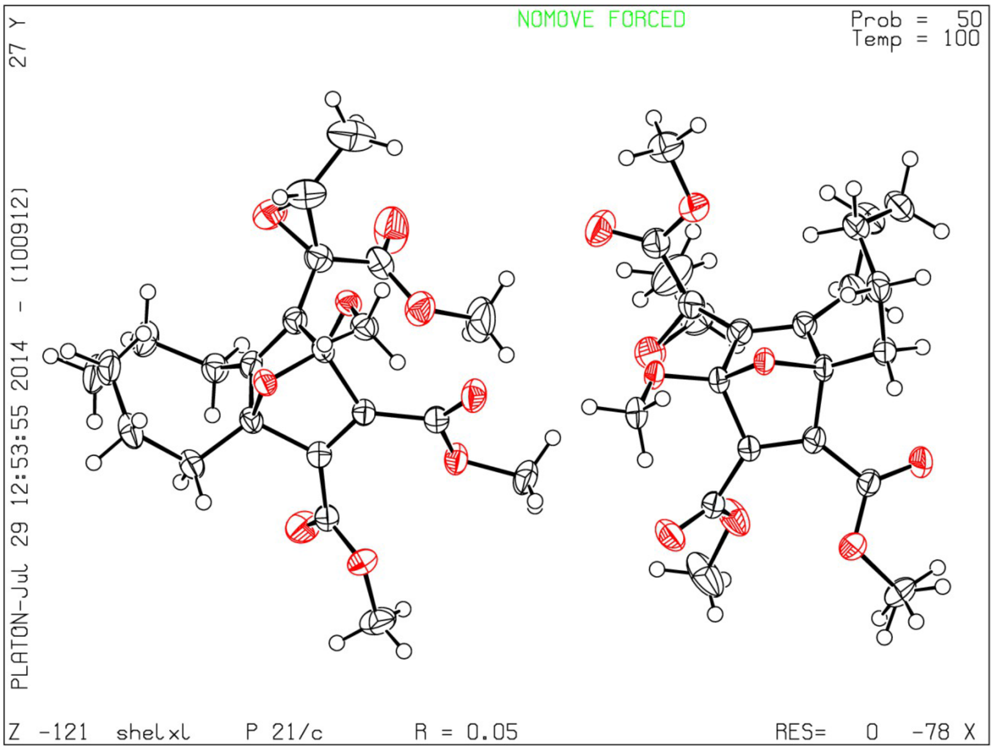

Figure S31. X-ray with atom labeling of **3b**.

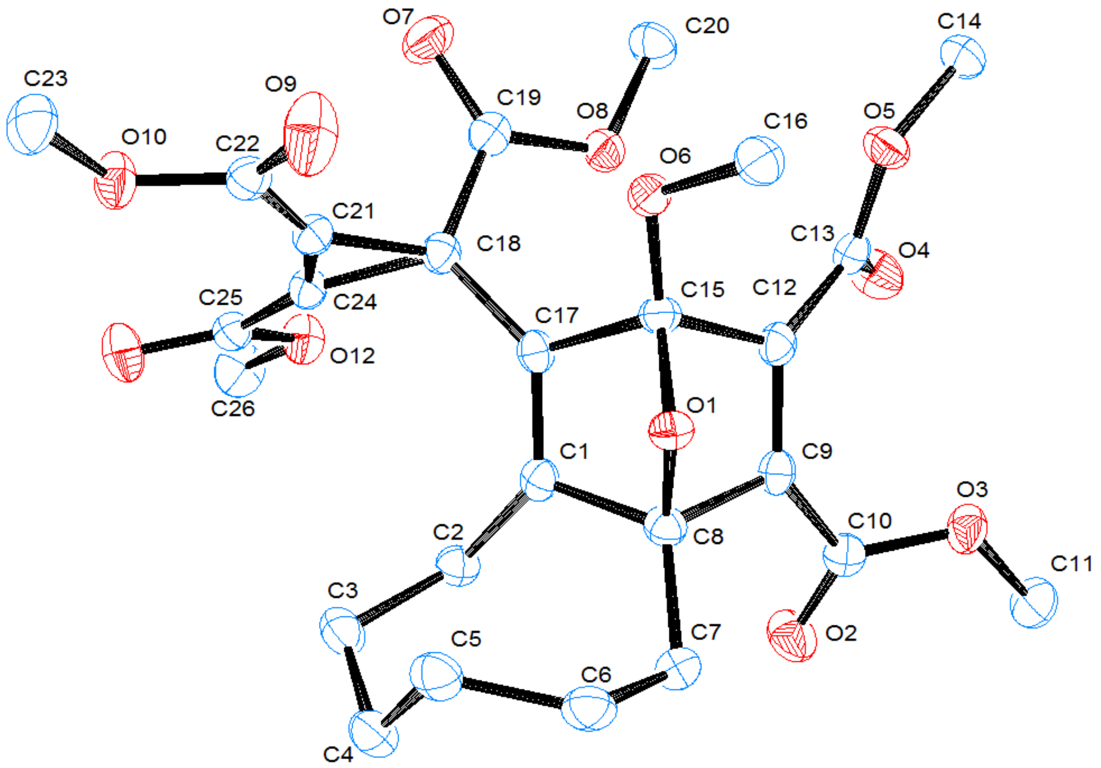

Figure S32. X-ray with atom labeling of **4a**.

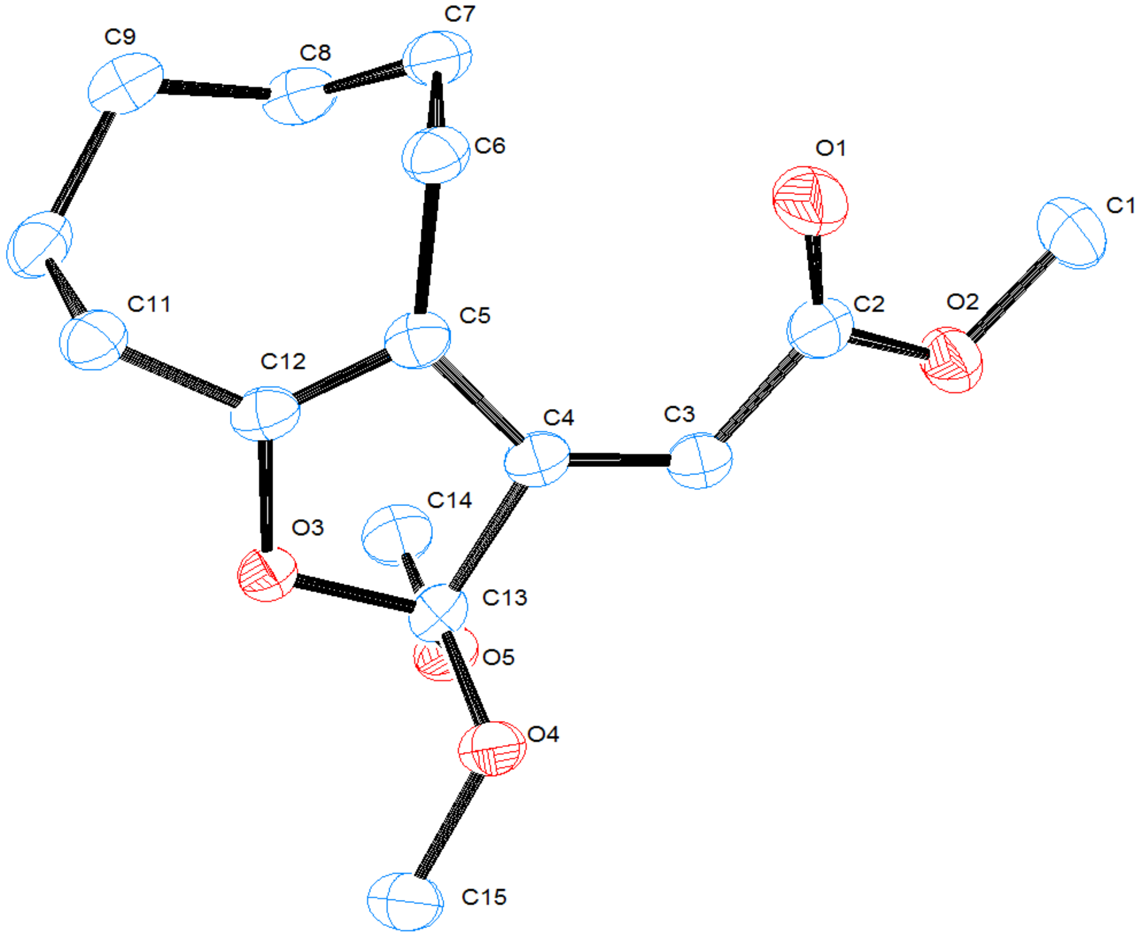

Figure S33. X-ray with atom labeling of **6a**.

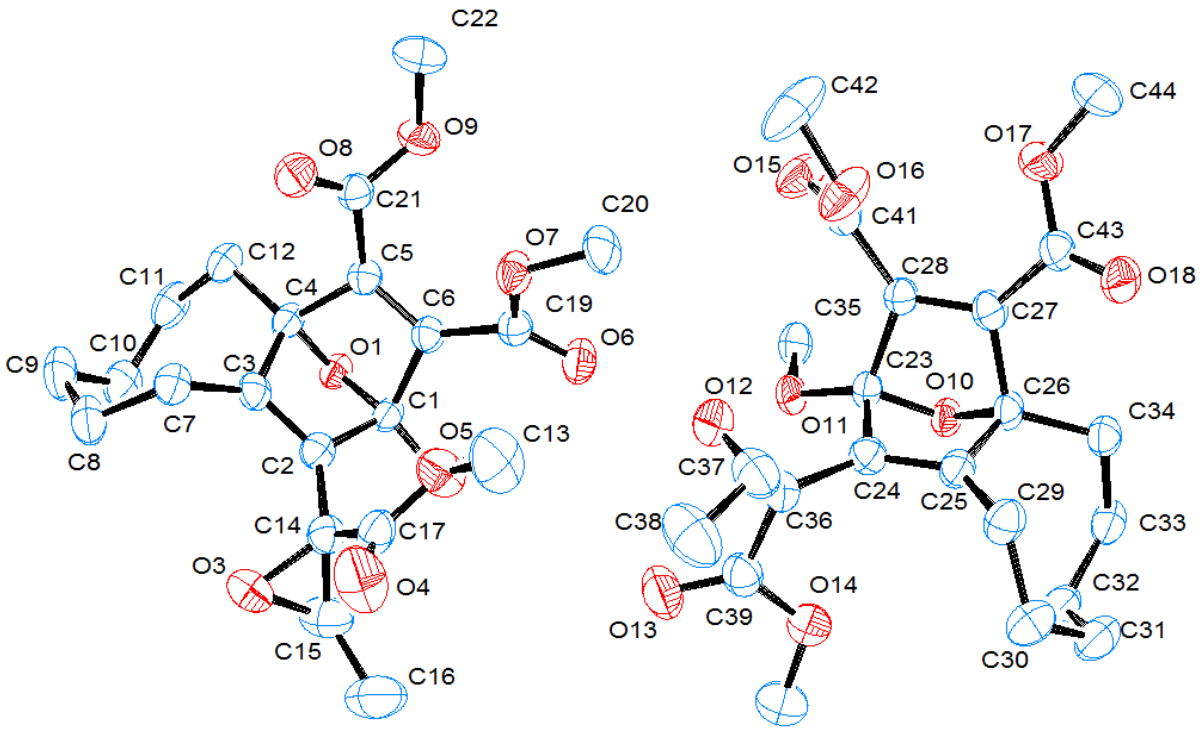

Supplement: Supplementary File 1 [file molecules-19-14022-s001.pdf]
